# PRKAB2 as a tumor suppressor in renal cell carcinoma: inhibiting mitophagy via the LRPPRC-PRKN/parkin interaction and cardiolipin biosynthesis

**DOI:** 10.1080/15548627.2026.2623985

**Published:** 2026-02-18

**Authors:** Kailei Chen, Yuanpeng Zhang, Hailong Ruan, Zhihao Wei, Keshan Wang, Qi Cao, Qi Wang, Zirui Dong, Yilong Wu, Hongmei Yang, Lei Liu, Yuenan Liu, Xiaoping Zhang

**Affiliations:** a Department of Urology, Union Hospital, Tongji Medical College, Huazhong University of Science and Technology, Wuhan, P. R. China; b Institute of Urology, Tongji Medical College, Huazhong University of Science and Technology, Wuhan, P. R. China; c Shenzhen Huazhong University of Science and Technology Research Institute, Shenzhen, P. R. China; d Department of Pathogenic Biology, School of Basic Medicine, Huazhong University of Science and Technology, Wuhan, P. R. China

**Keywords:** Cardiolipin, mitophagy, PRKAB2, renal cell carcinoma, TKI resistance

## Abstract

Renal cell carcinoma (RCC) is characterized by dysregulated lipid metabolism and a high propensity for developing resistance to targeted therapies. Mitophagy is a key process involved in the progression of various cancers, including RCC. Here, using *in vivo* genome-wide CRISPR screening, we identified PRKAB2 as a crucial tumor suppressor in RCC. Reduced PRKAB2 expression correlated with poor prognosis and aggressive clinical features, whereas overexpression of PRKAB2 markedly inhibited RCC cell proliferation, migration, invasion, tumor growth, and metastasis both *in vitro* and *in vivo*. Mechanistically, PRKAB2 overexpression inhibited mitophagy primarily through two distinct mechanisms. First, PRKAB2 enhanced the binding between LRPPRC and PRKN/parkin, competitively reducing PRKN’s interaction with PINK1 and thus suppressing ubiquitin-dependent mitophagy. Second, PRKAB2 promoted AMPK phosphorylation, which in turn suppressed SREBF1/SREBP1-mediated transcriptional activation of *CRLS1*, leading to decreased CRLS1 expression and reduced synthesis of cardiolipin, a lipid essential for mitophagy. Importantly, PRKAB2 overexpression significantly restored sensitivity to tyrosine kinase inhibitors (TKIs) in sunitinib-resistant RCC cells. Conversely, forced PRKN expression promoted resistance to these drugs, further implicating mitophagy as a key mechanism underlying TKI resistance. Depmap analysis confirmed the association between increased mitophagy and TKI resistance. Overall, our findings identify PRKAB2 as a critical tumor suppressor in RCC, regulating both protein-protein interactions and lipid metabolism to suppress mitophagy. Targeting PRKAB2-associated pathways may provide a promising therapeutic strategy to enhance treatment efficacy and overcome drug resistance in RCC.

**Abbreviations**: ACACA/ACC1: acetyl-CoA carboxylase alpha; AMPK: AMP-activated protein kinase; ATCC: American Type Culture Collection; ATP5F1A: ATP synthase F1 subunit alpha; BNIP3: BCL2 interacting protein 3; BNIP3L/NIX: BCL2 interacting protein 3 like; BRCA1: BRCA1 DNA repair associated; Cas: CRISPR-associated; CCCP: carbonyl cyanide m-chlorophenyl hydrazone; ccRCC: clear cell renal cell carcinoma; ChIP: chromatin immunoprecipitation; Co-IP: co-immunoprecipitation; COX4I1: cytochrome c oxidase subunit 4I1; CRISPR: clustered regularly interspaced short palindromic repeats; CRLS1: cardiolipin synthase 1; DNM1L/DRP1: dynamin 1 like; DOX: doxorubicin; FUNDC1: FUN14 domain containing 1; HSPA8: heat shock protein family A (Hsp70) member 8; HSPD1: heat shock protein family D (Hsp60) member 1; GO: gene ontology; IHC: immunohistochemistry; IMM: inner mitochondrial membrane; LDLR: low density lipoprotein receptor; m-SREBF1: mature sterol regulatory element binding transcriptional factor 1; LRPPRC: leucine rich pentatricopeptide repeat containing; MAP1LC3B: microtubule associated protein 1 light chain 3 beta; MFN1, mitofusin 1; MFN2: mitofusin 2; MTOR: mechanistic target of rapamycin kinase; OMM: outer mitochondrial membrane; OS: overall survival; PA: phosphatidic acid; PG: phosphatidylglycerol; PGS1: phosphatidylglycerophosphate synthase 1; PINK1: PTEN induced kinase1; PRKAA1/AMPKα1: protein kinase AMP-activated catalytic subunit alpha 1; PRKAA2/AMPKα2: protein kinase AMP-activated catalytic subunit alpha 2; PRKAB1/AMPKβ1: protein kinase AMP-activated catalytic subunit beta 1; PRKAB2/AMPKβ2: protein kinase AMP-activated non-catalytic subunit beta 2; PRKAG1/AMPKγ1: protein kinase AMP-activated non-catalytic subunit gamma 1; PRKN: parkin RBR E3 ubiquitin protein ligase; RCC: renal cell carcinoma; SASA: solvent-accessible surface areas; SUCLG1: succinate-CoA ligase GDP/ADP-forming subunit alpha; TCGA: The Cancer Genome Atlas; TKI: tyrosine kinase inhibitors; UCP1: uncoupling protein 1; ULK1: unc-51 like autophagy activating kinase 1; WCL: whole-cell lysate.

## Introduction

Renal cell carcinoma (RCC) is among the most prevalent malignancies of the urinary system with more than 430,000 new diagnoses in 2020 [1] and clear cell renal cell carcinoma (ccRCC) representing the predominant subtype [2]. Currently, radical surgical resection remains the primary treatment for localized RCC, while targeted therapy and immunotherapy are recommended for unresectable and advanced RCC [2]. However, RCC exhibits frequent resistance to targeted therapies and consequently poor prognosis, with a five-year survival below 10% [1]. Therefore, clarifying the molecular mechanisms underlying RCC from multiple perspectives and developing novel therapeutic strategies are critical for improving patient outcomes.

In recent years, mitochondrial dysfunction and metabolic reprogramming have emerged as critical factors in cancer progression. Certain cancer cells enhance mitochondrial biogenesis and mitophagy to adapt to metabolic stress, sustaining growth under nutrient-deprived and hypoxic conditions [3]. Notably, RCC demonstrates a pronounced reliance on lipid metabolism and oxidative phosphorylation compared to other malignancies [4]. Therefore, exploring the mechanisms underlying mitochondrial homeostasis is essential for understanding RCC’s metabolic dependencies and identifying potential therapeutic targets.

Mitophagy, a key mechanism for maintaining mitochondrial homeostasis, selectively removes damaged mitochondria to preserve metabolic balance and energy stability. In cancer, mitophagy plays complex roles, either facilitating tumor adaptation to metabolic stress or suppressing growth by eliminating reactive oxygen species [5,6]. The PINK1 (PTEN induced kinase 1)-PRKN/parkin (parkin RBR E3 ubiquitin protein ligase)-mediated ubiquitin-dependent pathway, a major regulator of mitophagy, is particularly relevant to tyrosine kinase inhibitors (TKIs) resistance in RCC [7]. Cardiolipin, a mitochondria-specific phospholipid predominantly located in the inner mitochondrial membrane (IMM), is increasingly recognized as an essential regulator of mitochondrial quality control, particularly mitophagy. Under mitochondrial stress, cardiolipin translocates to the outer mitochondrial membrane (OMM), functioning as a key signaling molecule that facilitates selective mitochondrial degradation. While mitophagy’s role in RCC progression and TKI resistance is increasingly recognized, its upstream regulatory signals remain largely unexplored. Understanding the key regulators orchestrating mitophagy, metabolic reprogramming, and treatment response is essential for developing innovative therapeutic strategies to modulate mitophagy and improve RCC treatment outcomes.

The AMP-activated protein kinase (AMPK) complex plays a crucial role in cellular energy homeostasis, lipid metabolism, and adaptation to metabolic stress [8]. Its function in cancer is dual-faceted. On the one hand, AMPK inhibits tumor cell proliferation by suppressing the MTOR (mechanistic target of rapamycin kinase) signaling pathway and glycolysis. On the other hand, under hypoxic or nutrient-deprived conditions, AMPK may promote fatty acid oxidation, thereby enhancing tumor cell survival [8]. Previous studies have demonstrated that AMPK promotes mitophagy in alcohol-induced liver injury by phosphorylating ULK1 (unc-51 like autophagy activating kinase 1) [9]. However, the role of AMPK in mitophagy regulation in RCC remains largely unknown, particularly in its impact on RCC metabolic adaptation and therapeutic response. Given the intricate interplay among AMPK signaling, mitophagy, and tumor metabolism, elucidating the regulatory mechanisms of AMPK-driven mitophagy in RCC may offer new therapeutic strategies for targeted RCC treatment.

Clustered regularly interspaced short palindromic repeats (CRISPR) and CRISPR-associated (Cas) proteins have revolutionized genome editing by enabling precise and efficient gene modifications [10]. CRISPR-based functional screening has emerged as a powerful tool for identifying key regulators of cancer progression. By introducing genome-wide perturbations, CRISPR screening allows systematic interrogation of gene functions, offering valuable insights into molecular mechanisms driving tumorigenesis and treatment resistance.

Our preliminary findings, utilizing *in vivo* genome-wide CRISPR screening and functional validation identified PRKAB2/AMPKβ2 (protein kinase AMP-activated non-catalytic subunit beta 2), the regulatory β2 subunit of AMPK complex, as a critical tumor suppressor in ccRCC. Mechanistically, PRKAB2 promotes the competitive binding of LRPPRC (leucine rich pentatricopeptide repeat containing) to PRKN to inhibit mitophagy and RCC progression. PRKAB2 also inhibits the synthesis of phosphatidylglycerol (PG) and cardiolipin, thereby suppressing mitophagy. Collectively, these results suggest PRKAB2 as a crucial regulator of mitophagy, highlighting its therapeutic potential to reverse TKI resistance and suppress tumor growth in RCC.

## Results

### Genome-wide in vivo CRISPR screening reveals a lipid metabolism-related regulatory network in RCC

**Figure 1.**
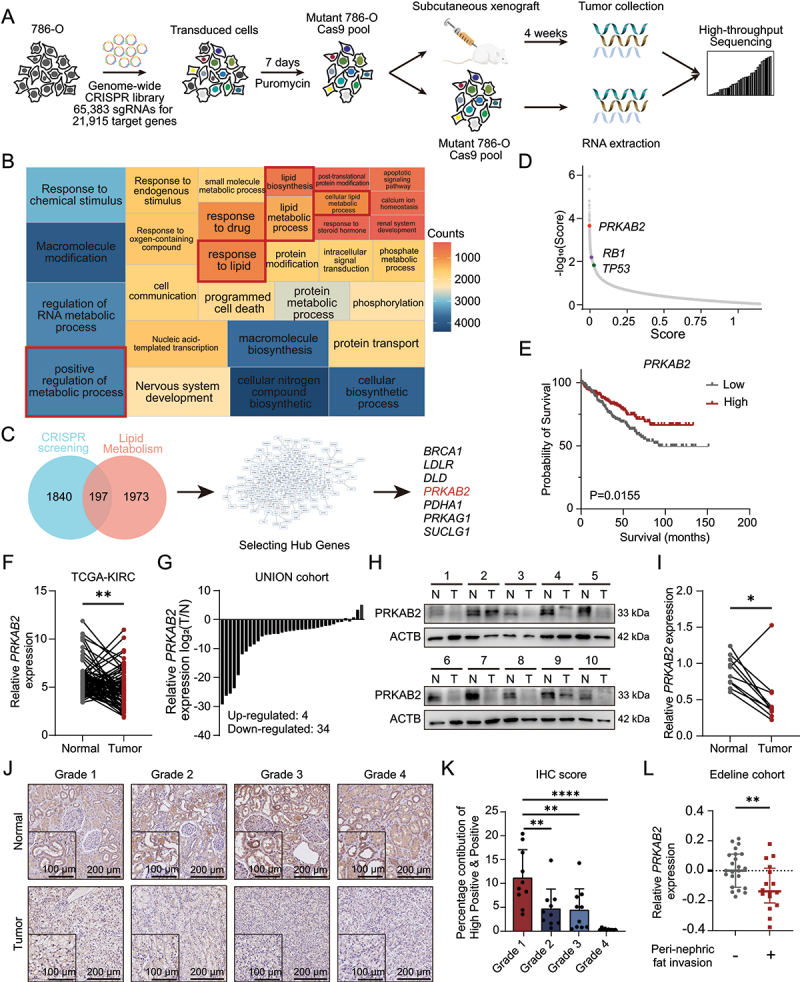
Genome-wide *in vivo* CRISPR screening identifies PRKAB2 as a potential tumor suppressor in RCC progression. (A) Schematic representation of the genome-wide *in vivo* CRISPR screening workflow. (B) Gene ontology (GO) analysis of candidate genes identified from genome-wide CRISPR screening. (C) Workflow illustrating the further selection of candidate genes based on lipid metabolic process-related pathways. (D) Distribution of enrichment scores for PRKAB2, RB1, and TP53 in the positive-selection screening. (E) Kaplan-Meier survival analysis of RCC patients with high versus low PRKAB2 expression in the TCGA-KIRC dataset. (F) PRKAB2 expression analysis in RCC tumor tissues compared with normal kidney tissues in the TCGA-KIRC dataset. (G) Comparison of *PRKAB2* mRNA expression in RCC tumor and adjacent normal kidney tissues from Union Hospital. (H) Western blot analysis of PRKAB2 protein expression in RCC tumor tissues versus adjacent normal kidney tissues from Union Hospital. (I) Quantitative grayscale analysis of PRKAB2 protein expression in RCC tumor and adjacent normal tissues from Union Hospital. (J) Immunohistochemical (IHC) analysis of PRKAB2 expression in RCC tumors of different pathological grades and adjacent normal tissues. (K) Quantification of PRKAB2 IHC scores across different pathological grades of RCC in Union Hospital samples. (L) PRKAB2 mRNA expression analysis in RCC patients with or without peri-nephric fat invasion in the Edeline cohort. *p < 0.05; **p < 0.01; ****p < 0.0001.

A key feature of malignant tumors is their invasive and robust tumor-forming ability, distinguishing them from immortalized epithelial cells. Thus, we introduced a genome-wide CRISPR library (GeCKOv2) [11] containing 65,383 sgRNAs targeting 21,915 human genes into ccRCC 786-O cells (Figure 1(A)). Cells underwent three independent biological replicates of lentiviral infection at a multiplicity of infection of 0.3, ensuring at least 500-fold coverage per sgRNA. Following puromycin selection for 7 days, 3 × 10^6^ cells were subcutaneously implanted into immunodeficient NSG mice, simulating *in vivo* tumor growth, while another 3 × 10^6^ cells were cryopreserved as baseline (*in vitro*) control samples (Cell Rep1, Rep2, Rep3). Tumor tissues were harvested after four weeks of *in vivo* selection (Figure S1A-B). High-throughput sequencing analysis of sgRNA distribution showed mapping ratios above 65% in both the baseline and tumor samples, indicating sufficient library coverage (Figure S1C). Gini indices for *in vitro* samples were low (0.1522–0.1678), demonstrating unbiased sgRNA distribution, whereas *in vivo* tumor samples exhibited increased Gini indices (0.4693–0.7140), reflecting selection pressure-induced enrichment (Figure S1D).

Next-generation sequencing coupled with gene ontology (GO) enrichment analysis revealed significant enrichment of lipid metabolism-related pathways, particularly “response to lipid”, “lipid metabolic process”, and “lipid biosynthetic process” (Figure 1(B)), suggesting their involvement in renal cell carcinoma progression. Our previous studies have demonstrated that lipid metabolism is essential for RCC progression [12]. Therefore, we initially identified genes demonstrating statistically significant associations (*p* < 0.05) with tumorigenesis and progression through *in vivo* functional screening. We then intersected these genes with a lipid metabolism-related gene set (Table S1), yielding a total of 197 genes (Figure 1(C,D)). Further CytoHubba analysis identified seven hub genes: *BRCA1* (BRCA1 DNA repair associated), *LDLR* (low density lipoprotein receptor), *SUCLG1* (succinate-CoA ligase GDP/ADP-forming subunit alpha), *DLD* (dihydrolipoamide dehydrogenase), *PDHA1* (pyruvate dehydrogenase E1 subunit alpha 1), *PRKAG1* (protein kinase AMP-activated non-catalytic subunit gamma 1), and *PRKAB2*. Kaplan-Meier survival analysis showed significant correlations between OS and three genes (*PRKAB2*, *DLD*, and *PDHA1*) (Figure 1E, S1E). Considering previously reported roles of PDHA1 and DLD in RCC, we selected PRKAB2 for further investigation [13,14]. A comprehensive analysis of the key genes identified in the CRISPR positive selection screen revealed that *PRKAB2*, along with classical tumor suppressor genes such as *RB1* and *TP53*, exhibited high selection scores. The presence of these well-established tumor suppressors further supports the robustness and reliability of our screening approach (Figure 1(D)). Survival data in Edline Cohort (GSE46699) further confirmed that RCC patients with lower PRKAB2 expression exhibited significantly shorter overall survival (OS) (Figure S1F). Expression analysis from The Cancer Genome Atlas (TCGA) revealed significantly lower PRKAB2 levels in tumor tissues compared to adjacent normal tissues (Figure 1(F)). qRT-PCR, western blot, and immunohistochemistry (IHC) analyses of RCC and adjacent normal tissues from the authors’ institute (Union cohort) revealed significantly reduced PRKAB2 expression in tumor tissues (Figure 1(G–J)). Notably, in normal kidney tissue, PRKAB2 expression was predominantly localized in renal tubular epithelial cells, which are the cellular origin of RCC (Figure 1(K)). This observation suggests that the downregulation of PRKAB2 may play a crucial role in the malignant transformation of renal tubular epithelial cells, contributing to RCC initiation. Furthermore, we observed that PRKAB2 expression decreased with increasing nuclear grade in ccRCC patients (Figure 1(K)). Consistently, data from the Edeline cohort showing that patients with lower PRKAB2 expression had a higher perinephric invasion risk (Figure 1(L)).

### PRKAB2 suppresses RCC progression in vitro and in vivo

**Figure 2.**
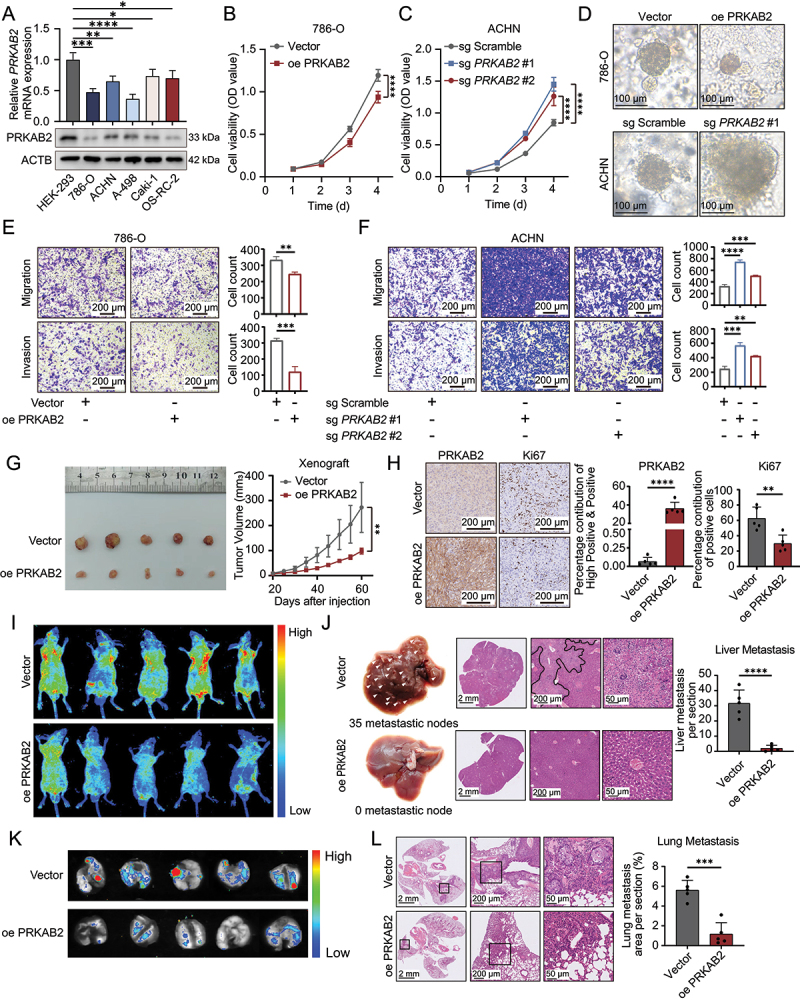
PRKAB2 suppresses RCC progression *in vitro* and *in vivo*. (A) PRKAB2 expression levels in HEK-293 and RCC cell lines, assessed by PCR and western blot analysis. (B and C) CCK-8 assay assessing the effect of PRKAB2 overexpression on RCC cell proliferation in 786-O (B) and ACHN (C) cell lines. (D) 3D culture assay evaluating the impact of PRKAB2 overexpression on RCC cell growth, scale bar: 100 μm. (E and F) Transwell assays assessing the effect of PRKAB2 overexpression (E) and knockout (F) on RCC cell migration and invasion. (G) Tumor growth monitoring in an RCC xenograft model established with PRKAB2-overexpressing cells. (H) IHC analysis of MKI67/Ki67 and PRKAB2 expression in RCC xenografts comparing PRKAB2-overexpressing and control tumors. (I) *In vivo* imaging to evaluate the effect of PRKAB2 overexpression on distant metastasis of RCC cells. (J) Quantification and statistical analysis of liver metastases following tail vein injection of RCC cells. (K) *In vivo* imaging assessing lung metastases after tail vein injection of RCC cells. (L) H&E staining of lung tissues to evaluate lung metastases following tail vein injection of RCC cells, with lung metastasis area quantification. *p < 0.05; **p < 0.01; ***p < 0.001; ****p < 0.0001.

We first validated PRKAB2 expression levels in common RCC cell lines and confirmed significantly reduced PRKAB2 levels at both mRNA and protein levels compared to normal renal cells (Figure 2(A)). Given the critical role of VHL (von Hippel-Lindau tumor suppressor) in RCC, we selected *VHL*-wild-type ACHN and *VHL*-mutant 786-O cells for further study. PRKAB2-overexpressing and *PRKAB2* knockout RCC cell lines were successfully established and validated (Figure S1K-L). CCK-8 and 3D organoid assays demonstrated that PRKAB2 overexpression significantly suppressed RCC cell proliferation (Figure 2(B–D), Figure S1M). Transwell assay demonstrated that PRKAB2 significantly suppressed invasion and migration of RCC cells (Figure 2E, S1O), whereas *PRKAB2* knockout markedly enhanced these malignant phenotypes (Figure 2F, S1N, S1P). The subcutaneous xenograft model demonstrated that PRKAB2 significantly suppressed RCC tumor growth *in vivo* (Figure 2(G)). IHC staining further revealed that Ki67 expression was markedly reduced in PRKAB2-overexpressing tumors, indicating a decline in proliferative capacity (Figure 2(H)). Similarly, the tail vein metastasis model showed that PRKAB2 significantly inhibited distant tumor metastasis (Figure 2(I)). Mice in the PRKAB2-overexpression group exhibited a substantial reduction in liver metastases (Figure 2(J)) as well as lung metastases (Figure 2(K,L)). Given that PRKAB2 is best known as a regulatory subunit of the AMPK complex [8]. we examined whether its tumor-suppressive function in RCC depends on AMPK signaling. Western blot analysis confirmed that PRKAB2 overexpression enhanced PRKAA/AMPKα (protein kinase AMP-activated catalytic subunit alpha) phosphorylation and modulated downstream lipid-related pathways, including phosphorylated ACACA/ACC1 (acetyl-CoA carboxylase alpha) as well as HMGCR (3-hydroxy-3-methylglutaryl-CoA reductase) and UCP1 (uncoupling protein 1) expression (Figure S1Q).

To test functional relevance, we silenced both *PRKAA1/AMPKα1* (protein kinase AMP-activated catalytic subunit alpha 1) and *PRKAA2/AMPKα2* (protein kinase AMP-activated catalytic subunit alpha 2) (Figure S1R). Knockdown markedly reduced PRKAA/AMPK phosphorylation and abolished PRKAB2-induced AMPK activation. CCK-8 assays and transwell assay showed that *PRKAA/AMPKα* silencing partially rescued the growth-inhibitory effect of PRKAB2, but not to the level of vector controls (Figure S1S-T). Interestingly, *PRKAA* silencing alone had little impact on baseline proliferation, likely due to compensatory pathways, consistent with previous reports in RCC models [15,16].

### PRKAB2 suppresses PINK1-PRKN -Mediated mitophagy

**Figure 3.**
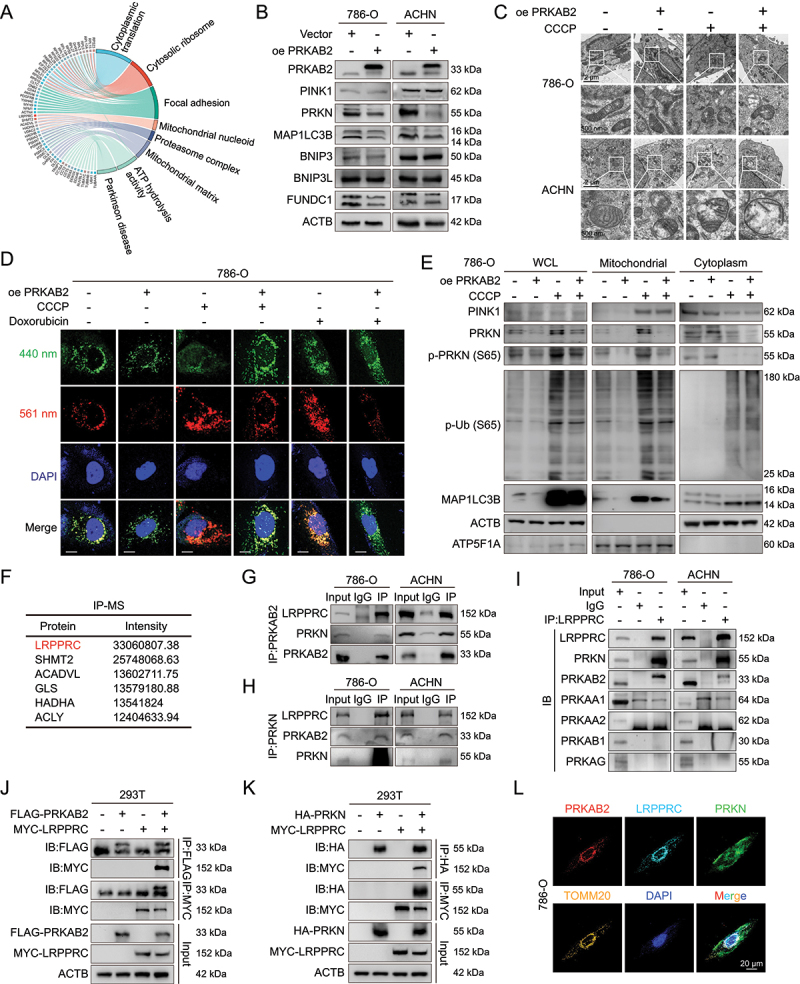
PRKAB2 regulates mitophagy by interacting with LRPPRC. (A) Gene ontology (GO) enrichment analysis of significantly enriched molecules identified from LC-MS/MS analysis. (B) Western blot analysis of mitophagy-related markers in RCC cells following PRKAB2 overexpression. (C) Transmission electron microscopy analysis of mitochondrial damage in RCC cells under different conditions. scale bars: 2 μm (low magnification), 500 nm (high magnification). (D) mt-Keima assay to assess mitophagy activation in RCC cells. Fluorescence channels: 440 nm (neutral pH) and 561 nm (acidic pH). scale bar: 20 μm. (E) Western blot of mitophagy-related markers in mitochondrial, cytosolic, and whole-cell lysate (WCL) fractions of RCC cells. (F) Ranking of mitochondrial-related proteins interacting with PRKAB2, identified by LC-MS/MS analysis. (G-I) Western blot analysis of PRKAB2 (G), PRKN (H), and LRPPRC (I) immunoprecipitations in RCC cells. (J) Co-IP assays in 293T cells co-expressing FLAG-PRKAB2 and MYC-LRPPRC, using FLAG or MYC affinity isolation. (K) Co-IP assays in 293T cells co-expressing HA-PRKN and MYC-LRPPRC, using HA-tag or MYC-tag affinity isolation. (L) Immunofluorescence staining in 786-O cells to assess the subcellular localization of PRKAB2, LRPPRC, PRKN, and TOMM20. scale bar: 20 μm. *p < 0.05; **p < 0.01; ***p < 0.001; ****p < 0.0001.

We next explored the mechanism by which PRKAB2 regulates RCC progression. Immunoprecipitation (IP) using PRKAB2 as bait followed by mass spectrometry and Gene Ontology (GO) enrichment analysis revealed significant enrichment of pathways related to mitochondrial nucleoid, mitochondrial matrix, and ATP hydrolysis activity (Figure 3(A)), indicating a strong association between PRKAB2 and mitochondrial function. Notably, GO analysis also identified enrichment of the Parkinson disease pathway and proteasome complex, both closely linked to the ubiquitin-proteasome system [17]. Because PINK1 and PRKN are well-known regulators of ubiquitin-dependent mitophagy [18], we hypothesized that PRKAB2 might modulate mitophagy in RCC cells. To investigate this, we assessed mitophagy-related proteins following PRKAB2 overexpression (Figure 3(B)). Although PINK1 expression remained unchanged, the expression of downstream proteins PRKN and MAP1LC3B/LC3B (microtubule associated protein 1 light chain 3 beta), particularly MAP1LC3B-II, a critical component of autophagosomal membranes [18], was significantly decreased. Conversely, *PRKAB2* knockout led to increased levels of PRKN and MAP1LC3B-II, suggesting that PRKAB2 negatively regulates mitophagy (Figure S2A).

Furthermore, upon treatment with the mitophagy inducer carbonyl cyanide m-chlorophenyl hydrazone (CCCP) [19] or doxorubicin (DOX) [20], mitochondrial damage and subsequent mitophagy were enhanced in RCC cells (Figure 3(C,D)). However, PRKAB2-overexpressing cells exhibited reduced mitophagy and increased accumulation of damaged mitochondria (Figure 3(C,D)). Mitochondrial fractionation showed that PRKAB2 overexpression in RCC cells reduced PRKN phosphorylation, mitochondrial translocation, and phosphorylated ubiquitin chains (Figure 3E, S2B). Conversely, *PRKAB2* knockout enhanced these markers of mitophagy (Figure S2C-D). Although total PINK1 levels remained largely unchanged after CCCP or DOX treatment, fractionation revealed increased mitochondrial-localized PINK1 under stress, consistent with pathway activation and previous reports [21]. In parallel, PRKAB2 overexpression led to accumulation of damaged mitochondria, reflected by elevated COX4I1/COXIV (cytochrome c oxidase subunit 4I1) and HSPD1/HSP60 (heat shock protein family D (Hsp60) member 1), whereas *PRKAB2* knockout decreased their levels (Figure S2E-F). Before mitophagy occurs, DNM1L/DRP1 (dynamin like 1) interacts with cardiolipin on the OMM, promoting mitochondrial fission into smaller fragments and thereby facilitating their engulfment by autophagosomes [22]. Thus, we evaluated key mitochondrial fusion and fission proteins (MFN1 [mitofusin 1], MFN2 [mitofusin 2], DNM1L), finding that PRKAB2 overexpression notably decreased DNM1L expression, suggesting suppression of mitochondrial fission (Figure S2G-H). Additionally, PRKAB2 did not affect BNIP3 (BCL2 interacting protein 3), BNIP3L/NIX (BCL2 interacting protein 3 like), or FUNDC1 (FUN14 domain containing 1), key regulators of ubiquitin-independent mitophagy [18], further indicating that PRKAB2 primarily regulates the PINK1-PRKN-dependent mitophagy signal (Figure 3B, S2A).

We next asked whether the inhibitory effect of PRKAB2 on mitophagy is dependent on the AMPK complex. Sequence analysis revealed that PRKAB2 contains a highly conserved region spanning amino acids 231–277 (Figure S3A-B), within which residues 248–271 of PRKAB/AMPKβ have been reported to mediate direct binding with the PRKAA [8]. To test this, we generated a mutant PRKAB2 construct (FLAG-PRKAB2Δ0–247) that fails to assemble into the AMPK complex (Figure S3C) and confirmed the loss of assembly by co-immunoprecipitation (co-IP) (Figure S3D). Interestingly, under CCCP-induced stress, the mutant still suppressed PRKN phosphorylation, mitochondrial translocation, and ubiquitin chain phosphorylation, although its inhibitory effect was weaker compared with wild-type PRKAB2 (Figure S3E-F).

### PRKAB2 facilitates competitive binding of LRPPRC to PRKN

To elucidate how PRKAB2 regulates mitophagy, we further analyzed PRKAB2-interacting proteins from the immunoprecipitation mass spectrometry results, identifying LRPPRC as the most abundant mitochondrial protein associated with PRKAB2 (Figure 3F, S3G, Table S2). Co-IP experiments in RCC cell lines (786-O and ACHN) confirmed direct interactions between PRKAB2 and LRPPRC (Figure 3G–I). LRPPRC was previously reported to inhibit mitophagy by binding PRKN [23], which is also confirmed here in RCC cells (Figure 3G,I). Given that PRKAB2 is the β2 subunit of AMPK, we tested whether LRPPRC associates with other subunits. Co-IP revealed a specific interaction with PRKAB2/AMPKβ2, but not with PRKAA1, PRKAA2, PRKAB1/AMPKβ1, or PRKAG1/AMPKγ, PRKAG2 and PRKAG3 (Figure 3I). Further validation in 293T cells demonstrated interactions between exogenous PRKAB2 and LRPPRC as well as LRPPRC and PRKN (Figure 3J,K). Finally, confocal imaging on immunofluorescence staining in 786-O and ACHN cells confirmed co-localization of PRKAB2, LRPPRC, and PRKN within mitochondria, further supporting the functional interplay of these proteins in regulating mitophagy (Figure 3L, S3H).

**Figure 4.**
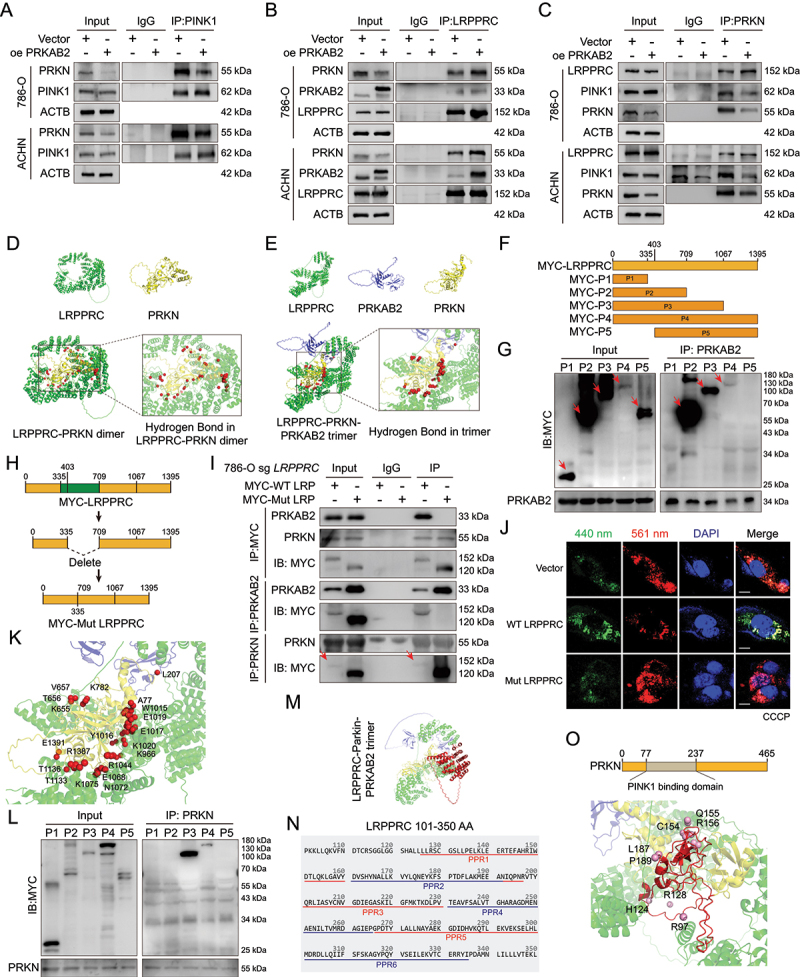
PRKAB2 modulates PRKN-LRPPRC interactions and stabilizes the mitophagy regulatory complex. (A) Co-IP and western blot analysis of PRKN interaction with PINK1 in control (vector) and PRKAB2-overexpressing RCC cells. (B) Co-IP and western blot analysis of PRKN and PRKAB2 interaction with LRPPRC in control and PRKAB2-overexpressing RCC cells. (C) Co-IP and western blot analysis of PINK1 and LRPPRC interaction with PRKN in control and PRKAB2-overexpressing RCC cells. (D and E) AlphaFold- and PyMOL-based structural predictions of hydrogen bond interactions at the PRKN-LRPPRC binding interface without (D) or with (E) PRKAB2. (F) Schematic of LRPPRC truncation constructs based on UniProt domain annotations and AlphaFold structural predictions. (G) Co-IP and western blot analysis identifying LRPPRC fragments that bind PRKAB2. (H) Schematic of LRPPRC mutant constructs. (I) Co-IP and western blot analysis of interactions between WT LRPPRC or mutant LRPPRC (labeled as MYC-Mut LRPPRC) with PRKAB2 and PRKN, using MYC, PRKAB2, or PRKN as bait proteins. MYC-WT LRP: refer to MYC-WT LRPPRC; MYC-Mut LRP: refer to MYC-Mut LRPPRC. (J) mt-Keima assay assessing the regulatory effects of WT and mutant LRPPRC on mitophagy under CCCP-induced mitochondrial stress in RCC cells. Fluorescence channels: 440 nm (neutral pH) and 561 nm (acidic pH). scale bar: 20 μm. (K) Predicted key amino acid residues at the PRKN-LRPPRC interface based on AlphaFold and PyMOL modeling. (L) Co-IP and western blot analysis identifying LRPPRC fragments that bind PRKN. (M) Predicted three-dimensional structure of the PRKAB2-LRPPRC-PRKN trimeric complex. Green: LRPPRC (404–1067 AA), blue: PRKAB2, yellow: PRKN, red: LRPPRC (1–403 AA). (N) Amino acid sequence alignment of LRPPRC (101–350 AA) highlighting its PPR domain. (O) Structural representation of critical PRKN residues within the PINK1-binding domain that also interact with the PRKAB2-LRPPRC complex.

Mechanistic analysis revealed that overexpression of PRKAB2 decreased the interaction between PRKN and PINK1 (Figure 4A). Given previous report [23] and our co-IP mass spectrometry results, we hypothesized that LRPPRC might compete with PINK1 for PRKN binding. Upon PRKAB2 overexpression, co-IP assays were performed using LRPPRC to affinity isolate PRKN and using PRKN to affinity isolate LRPPRC and PINK1. It revealed that in PRKAB2-overexpressing cells, the interaction between LRPPRC and PRKN was significantly enhanced, whereas the binding between PRKN and PINK1 was markedly reduced (Figure 4B,C). Structural predictions using AlphaFold revealed a reduction in the interface surface area between LRPPRC and PRKN in the LRPPRC-PRKN-PRKAB2 trimeric complex compared to the LRPPRC-PRKN dimer (17.751 vs. 19.166 Å^2^). Additionally, the number of interfacial hydrogen bonds decreased from 55 to 45 pairs in the trimeric complex (Figure 4D,E). It suggested that PRKAB2 might induce conformational changes in LRPPRC, promoting stronger interactions with PRKN and reducing PRKN’s availability to bind PINK1. To validate the role of PRKAB2 in promoting LRPPRC-PRKN competition, we used AlphaFold to predict PRKAB2-contacting residues of LRPPRC within 3.5 Å, mainly clustered in the 335–709 aa region (D367, Q538, R541, S542, D590, N621, R624, K649, L651; Figure S3I-J). Truncation analysis showed that PRKAB2 interacts with LRPPRC fragments containing this region, identifying 335–709 aa as essential for binding (Figure 4F,G). Deletion of this segment (MYC-mutant LRPPRC) abolished PRKAB2 binding in co-IP assays (Figure 4H, S3K).

Endogenous *LRPPRC* was knocked out in 786-O cells and rescued with WT LRPPRC or mutant LRPPRC. Co-IP revealed that PRKN bound both forms, whereas PRKAB2 interacted only with WT LRPPRC (Figure 4(I)). Upon CCCP or DOX treatment, WT LRPPRC restored mitophagy suppression, but the mutant did not (Figure 4J, S3L), indicating that the 335–709 AA region is required for PRKAB2 binding and for PRKAB2-mediated reinforcement of the LRPPRC-PRKN interaction.

To pinpoint critical interaction domains, we further analyzed the predicted binding sites and identified several essential residues mediating LRPPRC-PRKN interactions, including K782, K966, W1015, Y1016, E1017, E1019, K1020, R1044, E1068, N1072, K1075, T1133, T1136, R1387, and E1391 (primarily localized between residues 709-1395AA), as well as residues A77, K655, K656, and V657 (localized between residues 1-709AA) (Figure 4(K)). Truncated fragments containing residues 0-1067AA (P3) or 1-1395AA (P4) efficiently bound PRKN, confirming that LRPPRC’s 0-1067AA region was essential for PRKN interaction (Figure 4(L)). Conversely, the truncated protein P5 containing most key residues but lacking the N-terminal region (0-403AA) did not bind PRKN, suggesting the importance of the 0-403AA region for proper folding and stability. Structural analysis indicated the 0-403AA segment included multiple α-helical repeats forming critical PPR domains (Figure 4(M,N)). Furthermore, an N-terminal intrinsically disordered region (about 50 amino acids) may provide flexibility necessary for protein folding, suggesting its removal potentially disrupts structural integrity [24].

Additionally, analysis of PRKN residues involved in LRPPRC binding identified M1, D18, D20, E28, K32, R97, R128, H124, C154, Q155, R156, L187, P189, C241, T242, D243, and N295 as key residues mediating LRPPRC-PRKAB2-PRKN binding. Notably, eight residues (R97, R128, H124, C154, Q155, R156, L187, P189) were precisely located within the PINK1-binding domain of PRKN. (Figure 4(O)), strongly supporting a competitive interaction model between LRPPRC and PINK1 for PRKN binding.

### PRKN degradation via chaperone-mediated autophagy

PRKAB2 overexpression reduced total PRKN protein levels without affecting its mRNA expression (Figure 3A, S3M), indicating post-transcriptional regulation. Cycloheximide chase assays showed accelerated PRKN degradation (Figure S3N). While PRKN is often degraded via the proteasome [25], only chloroquine, but not MG132, restored PRKN levels in PRKAB2-overexpressing cells (Figure S3O), suggesting involvement of the autophagy-lysosome pathway. Moreover, 3-MA treatment failed to reverse PRKN reduction (Figure S3P), implicating chaperone-mediated autophagy (CMA) rather than macroautophagy. Consistently, co-IP confirmed PRKN-HSPA8/Hsc70 (heat shock protein family A (Hsp70) member 8) interaction (Figure S3Q), supporting CMA-mediated degradation [26]. These findings suggest that PRKAB2 decreases PRKN protein levels primarily by promoting CMA-dependent degradation.

### Knockout of LRPPRC partially reverses PRKAB2-mediated inhibition of mitophagy and RCC progression

**Figure 5.**
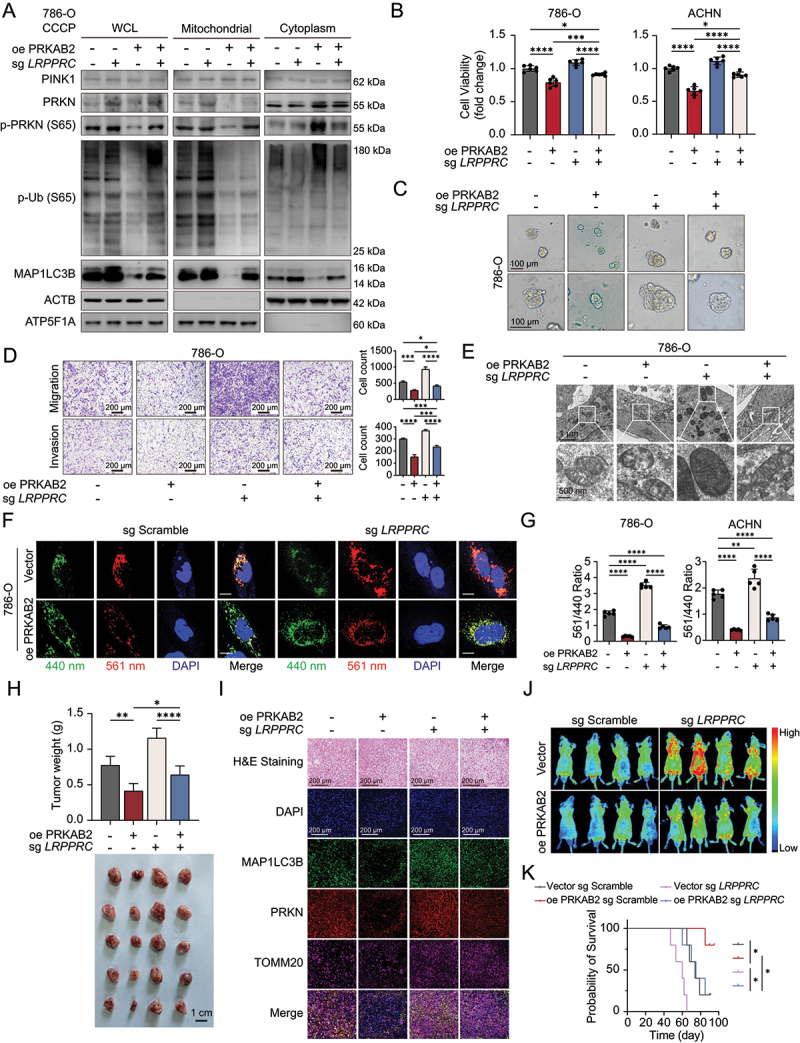
LRPPRC depletion partly rescue the effect of PRKAB2 overexpression. (A) Mitochondrial fractionation and western blot analysis of ubiquitin-dependent mitophagy markers in RCC cells following treatment with 10 μM CCCP for 2 h. (B) CCK-8 assay evaluating the proliferative capacity of RCC cells after 72 h of culture by measuring absorbance at 450 nm. (C) 3D culture assay assessing the proliferative potential of 786-O cells, as indicated. (D) Transwell invasion and migration assays in 786-O cells overexpressing PRKAB2 or knockout of *LRPPRC*. (E) Transmission electron microscopy analysis of mitochondrial damage in 786-O cells following treatment with 20 μM CCCP for 2 h, as indicated. scale bars: 1 μm (low magnification), 500 nm (high magnification). (F) mt-Keima assay to assess mitophagy in 786-O cells following treatment with 10 μM CCCP for 2 h. Fluorescence channels: 440 nm (neutral pH) and 561 nm (acidic pH). scale bar: 20 μm. (G) Quantification of mt-Keima fluorescence images, with statistical comparisons performed using one-way ANOVA. (H) Representative images of subcutaneous xenograft tumors and tumor weight comparisons in nude mice implanted with RCC cells, as indicated. (I) Immunofluorescence staining of xenograft tumors. scale bar: 200 μm. (J) Representative *in vivo* imaging of distant metastases in mice injected with RCC cells, as indicated (*N* = 5). (K) Kaplan-Meier survival analysis of mice in the metastasis model (*N* = 5) implanted with RCC cells, as indicated. *p < 0.05; **p < 0.01; ***p < 0.001; ****p < 0.0001.

To further validate the role of LRPPRC in PRKAB2-mediated mitophagy regulation, *LRPPRC* was knocked out in RCC cells using the CRISPR-Cas9 system (Figure S4A). We observed enhanced activation of mitophagy, as indicated by increased expression of PINK1-PRKN pathway components in *LRPPRC* knockout cells (Figure 5A, S4B). Importantly, *LRPPRC* depletion partially reversed the suppressive effects of PRKAB2 overexpression on RCC cell proliferation (Figure 5(B,C)), migration, and invasion (Figure 5D, S4C). Transmission electron microscopy analysis revealed that *LRPPRC* knockout accelerated mitophagy clearance of damaged mitochondria, which was further confirmed by mt-Keima assay (Figure 5E–G, S4D-F). Both subcutaneous xenograft assays and metastasis models demonstrated that *LRPPRC* knockout could partially reverse the tumor-suppressive effects induced by PRKAB2 *in vivo* (Figure 5H–K, S4G). Furthermore, immunofluorescence staining of the subcutaneous xenografts revealed that LRPPRC deletion also reversed the inhibitory effect of PRKAB2 on mitophagy (Figure 5I, S4G). Collectively, these findings indicate that LRPPRC is a critical mediator of PRKAB2’s inhibition of mitophagy and RCC progression. However, *LRPPRC* knockout did not fully rescue the suppressive effects of PRKAB2 on mitophagy and RCC progression, indicating that other pathways may contribute to its regulatory role.

### PRKAB2 suppresses mitophagy by downregulating CRLS1-driven cardiolipin biosynthesis

**Figure 6.**
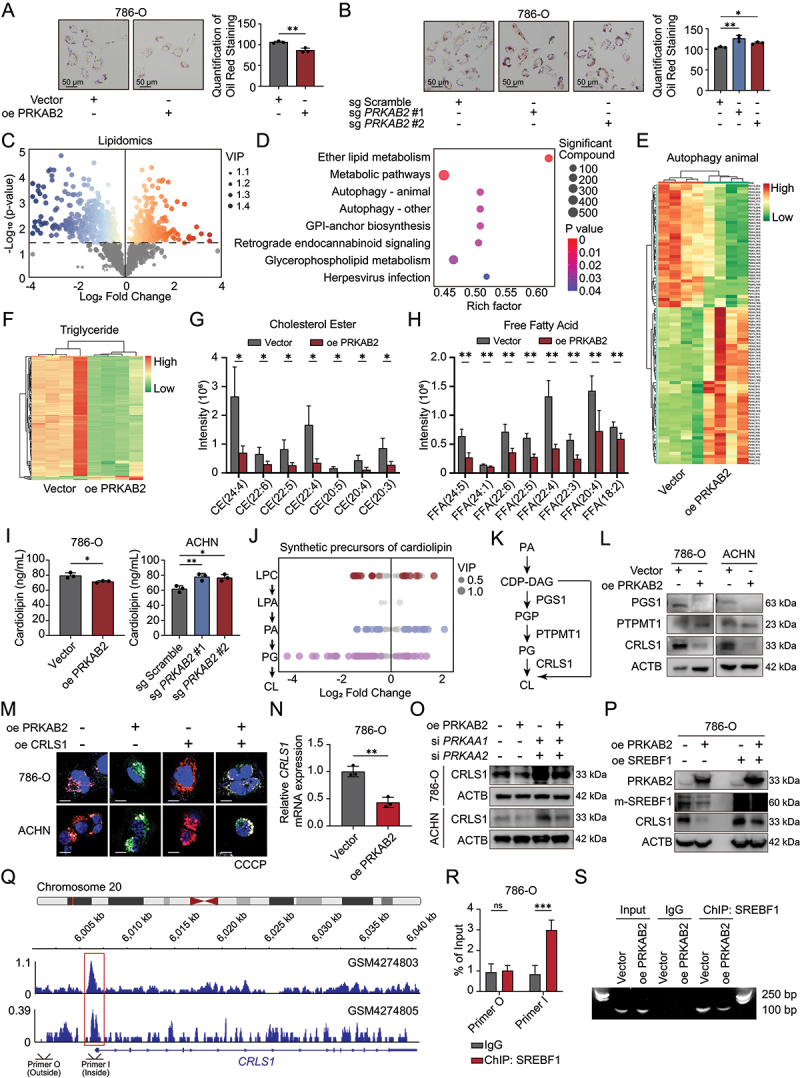
PRKAB2 regulates lipid metabolism and cardiolipin synthesis in RCC cells. (A and B) Oil red O staining of 786-O cells with PRKAB2 overexpression (A) or *PRKAB2* knockout (B) scale bar: 50 μm. (C) Broad-spectrum targeted lipidomics analysis revealing changes in lipid composition following PRKAB2 overexpression. (D) Gene ontology (GO) analysis of differentially regulated lipids identified in PRKAB2-overexpressing cells from lipidomics analysis. (E and F) Changes in autophagy-related lipids (E) and triglycerides (F) in PRKAB2-overexpressing RCC cells, based on lipidomics analysis. (G and H) Alterations in representative cholesteryl esters (G) and free fatty acids (H) in PRKAB2-overexpressing RCC cells, identified by lipidomics profiling. (I) Quantification of cardiolipin levels in PRKAB2-overexpressing or knockout RCC cells. (J) Changes in cardiolipin precursor lipids following PRKAB2 overexpression, based on lipidomics analysis. LPC, lysophosphatidyl choline; LPA, lysophosphatidic acid; PA, phosphatidic acid; PG, phosphatidylglycerol; CL, cardiolipin. (K) Schematic representation of intracellular cardiolipin biosynthesis pathways. (L) Western blot analysis of key enzymes involved in cardiolipin biosynthesis following PRKAB2 overexpression. (M) mt-Keima assay to assess mitophagy in 786-O cells following treatment with 10 μM CCCP for 2 h. Fluorescence channels: 440 nm (neutral pH) and 561 nm (acidic pH). scale bar: 20 μm. (N) PCR analysis of *CRLS1* expression in PRKAB2-overexpressing and vector control groups. (O) Western blot analysis of CRLS1 protein expression levels, as indicated. (P) Western blot analysis of m-SREBF1 (mature sterol regulatory element binding transcription factor 1) protein expression levels, as indicated. (Q) ChIP-seq results indicating the potential binding intensity of SREBF1 to the *CRLS1* promoter region. (R) qPCR analysis of amplified products from SREBF1 ChIP using different primer sets. (S) DNA gel electrophoresis showing the amplification products of SREBF1 ChIP with primer I, as indicated. *p < 0.05; **p < 0.01; ***p < 0.001; ns, not statistically significant.

Since *LRPPRC* knockout only partially reversed the inhibition of mitophagy caused by PRKAB2 overexpression, we reasoned that additional mechanisms must be involved. Lipid metabolism not only plays a crucial role in RCC progression but also directly influences mitophagy. Considering that PRKAB2 is a regulatory subunit of the AMPK complex, a key modulator of cellular lipid metabolism, we next explored whether PRKAB2 might suppress mitophagy through reprogramming lipid metabolic pathways. Therefore, we examined lipid metabolism alterations following PRKAB2 alterations. Overexpression of PRKAB2 significantly decreased cholesterol and triglyceride accumulation in RCC cells, while *PRKAB2* knockout increased these lipids (Figure 6A,B, Figure S5A-B). Broad-spectrum targeted lipidomics analysis revealed 410 lipid species significantly reduced and 259 increased after PRKAB2 overexpression (Figure 6C, Table S3). Kyoto Encyclopedia of Genes and Genomes enrichment analysis of these altered lipids highlighted significant involvement in autophagy, GPI-anchor biosynthesis, and ether lipid metabolism (Figure 6(D,E)), suggesting a lipid-mediated mechanism for PRKAB2 in regulating mitophagy.

Detailed lipidomic profiling showed that PRKAB2 overexpression markedly reduced triglycerides, cholesterol esters, and free fatty acids, the major components of lipid droplets (Figure 6(F–H)). Given cardiolipin’s critical role in mitophagy initiation, we analyzed cardiolipin levels and found that PRKAB2 overexpression substantially reduced cellular cardiolipin content, whereas *PRKAB2* knockout significantly increased its levels (Figure 6I, S5C). Cardiolipin synthesis, mainly driven by phosphatidylglycerol (PG) metabolism in the IMM, was further examined [27]. Lipidomics analysis demonstrated that PRKAB2 overexpression significantly decreased multiple phosphatidylglycerol (PG) species without affecting upstream phosphatidic acid (PA) levels (Figure 6(J,K)). These results suggest PRKAB2 may influence cardiolipin synthesis by specifically modulating PG or cardiolipin biosynthesis enzymes. Correspondingly, expression analysis showed decreased levels of PGS1 (phosphatidylglycerophosphate synthase 1) and CRLS1 (cardiolipin synthase 1) (Figure 6(L)). PGS1 is a key enzyme in cardiolipin biosynthesis, catalyzing the conversion of PA and cytidine diphosphate-glycerol into phosphatidylglycerophosphate, which is subsequently dephosphorylated to generate PG, a precursor for cardiolipin synthesis [28]. CRLS1, a mitochondrial inner membrane-specific cardiolipin synthase, then facilitates the final step of cardiolipin biosynthesis by catalyzing the reaction between PG and PA, thereby playing a crucial role in maintaining mitochondrial membrane stability and function [28]. We overexpressed CRLS1 on PRKAB2-overexpressing RCC cells and observed restored cardiolipin levels (Figure S5D-E). Mt-keima assay demonstrated that CRLS1 effectively reversed the inhibitory effect of PRKAB2 overexpression on mitophagy induced by CCCP (Figure 6M, S5F), confirming CRLS1 and cardiolipin as critical mediators through which PRKAB2 suppresses mitophagy. To further elucidate the mechanism by which PRKAB2 downregulates CRLS1, *CRLS1* mRNA levels were examined in RCC cells. PRKAB2 overexpression reduced *CRLS1* mRNA, whereas *PRKAB2* knockout increased its transcription (Figure 6N, S5G-H). Silencing of *PRKAA* reversed the inhibitory effect of PRKAB2 on CRLS1 expression (Figure 6O, S5I), indicating an AMPK-dependent mechanism. As SREBF1 is a canonical transcription factor negatively regulated by AMPK [29], its role in CRLS1 regulation was assessed. SREBF1 overexpression fully restored CRLS1 expression suppressed by PRKAB2 (Figure 6P, S5J-K). ChIP (Chromatin Immunoprecipitation)-seq data further revealed a potential SREBF1-binding peak near the CRLS1 promoter [30] (Figure 6(Q)). ChIP-qPCR confirmed significant SREBF1 occupancy at this site, which was markedly reduced under PRKAB2 overexpression (Figure 6R,S, S5L-M). These results suggest that PRKAB2 inhibits cardiolipin synthesis, and consequently mitophagy, by repressing SREBF1-dependent transcription of *CRLS1*.

### Dual loss of LRPPRC and PRKAA abolishes PRKAB2 effects

CCK-8 and Transwell assays demonstrated that either *LRPPRC* knockout or *PRKAA* silencing alone only partially rescued PRKAB2-mediated suppression of cell proliferation and migration, whereas combined perturbation fully restored these phenotypes (Figure S6A-C). Consistently, mt-Keima assays showed that PRKAB2-induced inhibition of mitophagy was only partially alleviated by individual *LRPPRC* knockout or *PRKAA* silencing, but was completely reversed when both were applied simultaneously (Figure S6D-E). These findings indicate that PRKAB2 exerts its tumor-suppressive effects through both promoting LRPPRC-PRKN interaction and modulating AMPK-dependent signaling.

### PRKAB2 enhances sensitivity to TKIs in drug-resistant RCC cells by inhibiting mitophagy

**Figure 7.**
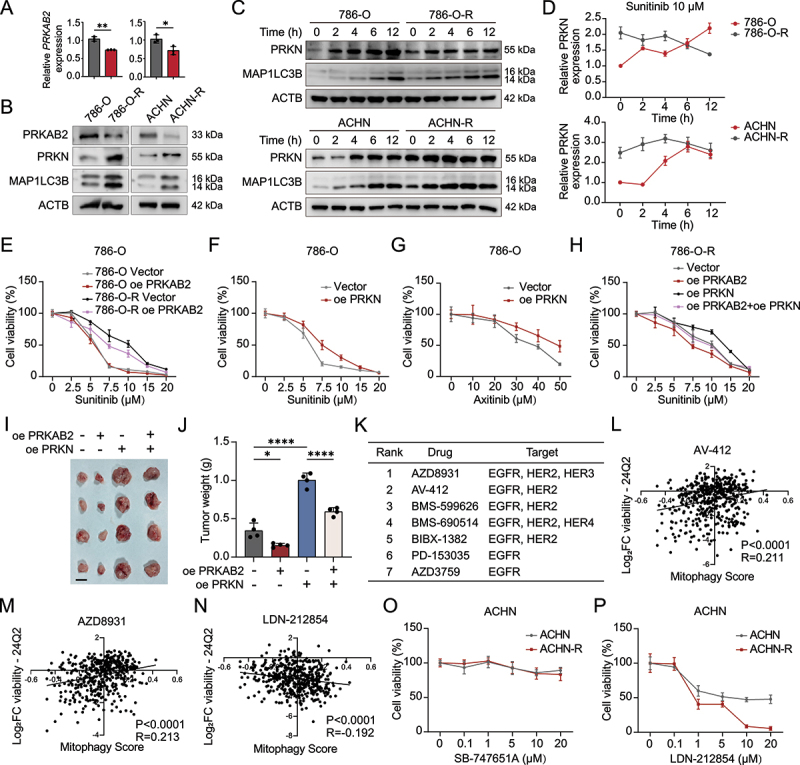
PRKAB2 modulates mitophagy and TKI sensitivity in RCC, with potential therapeutic implications. (A) PCR analysis of *PRKAB2* in parental and sunitinib-resistant 786-O and ACHN cell lines. (B) Western blot analysis of PRKAB2 and key mitophagy markers in parental and sunitinib-resistant 786-O and ACHN cell lines. (C) Western blot analysis of mitophagy marker changes in parental and sunitinib-resistant 786-O and ACHN cell lines after 10 μM sunitinib treatment for 2–12 h. (D) Line graph showing grayscale quantification of mitophagy marker changes from (C). (E) CCK-8 assay measuring sunitinib IC50 in parental and sunitinib-resistant 786-O cell lines overexpressing PRKAB2. (F-G) CCK-8 assay measuring IC50 values of sunitinib (F) and axitinib (G) in 786-O cells overexpressing PRKN. (H) CCK-8 assay measuring sunitinib IC50 in sunitinib-resistant 786-O cells overexpressing PRKAB2 or PRKN. (I and J) *In vivo* RCC xenograft model: Representative tumor images (I) and tumor weight comparisons (J) in sunitinib-resistant 786-O xenografts treated with 60 mg/kg sunitinib by oral gavage, as indicated. scale bar: 1 cm. (K) DepMap analysis identifying the top seven drugs whose sensitivity is most negatively correlated with mitophagy scores, along with their corresponding targets. (L-N) Scatter plots from DepMap database showing correlations between mitophagy scores and drug sensitivity for AV-412 (L), AZD8931 (M), and LDN-212854 (N). (O and P) CCK-8 assay measuring IC50 values of SB-747651A (O) and LDN-212854 (P) in parental and sunitinib-resistant ACHN cells. *p < 0.05; **p < 0.01; ****p < 0.0001.

Given that mitophagy has been implicated in TKI resistance [7], we explored whether PRKAB2 affects TKI sensitivity via modulation of mitophagy in RCC cells. Initially, we examined the expression of mitophagy-related proteins in our previously established sunitinib-resistant RCC cell lines (Figure S7A-B). Compared with parental cells, resistant cells displayed elevated expression of PRKN and MAP1LC3B-II, indicating higher mitophagy activity (Figure 7(A,B)). Sunitinib treatment (10 µM, 2–12 h) robustly induced mitophagy in parental RCC cells, but elicited only a modest response in resistant cells, potentially due to their higher basal autophagic activity (Figure 7(C,D)). Next, we overexpressed PRKAB2 or PRKN in both parental and resistant RCC cells (Figure S7C). In parental cells, PRKAB2 overexpression did not significantly enhance sunitinib sensitivity, possibly due to their inherent high sensitivity to the drug. However, PRKAB2 overexpression significantly restored sensitivity to sunitinib in resistant cells (Figure 7E, S7D). Conversely, PRKN promoted resistance to sunitinib and axitinib in parental RCC cells (Figure 7F,G, S7E-F), supporting the notion that mitophagy activation drives TKI resistance. Additionally, co-overexpression experiments indicated that PRKN reversed the increased sunitinib sensitivity mediated by PRKAB2 overexpression in resistant RCC cells (Figure 7H, S7G). These findings were also validated *in vivo*, where PRKAB2 overexpression enhanced the efficacy of sunitinib, while PRKN reversed this effect (Figure 7(I,J)).

We further explored the correlation between mitophagy and drug sensitivity using data from the DepMap database. Correlation analyses revealed a strong negative relationship between mitophagy scores and sensitivity to EGFR and TKI pathway inhibitors, as indicated by log_2_FC viability, where a more negative value reflects higher drug sensitivity (Figure 7K–M, S7H-J). Notably, the top seven drug targets with the most negative Pearson correlation coefficients were all associated with the TKI signaling pathway (Figure 7(K)). Given that higher mitophagy scores were associated with increased resistance to TKI inhibitors, we sought to identify compounds that selectively target cells with high mitophagy activity. Through correlation analyses, we identified two compounds, LDN212854 and SB-747651A, whose log_2_FC viability values were positively correlated with mitophagy scores (Pearson *R* > 0.2; Figure 7N, S7K). This indicates that cells with higher mitophagy activity exhibited increased sensitivity to these compounds. CCK-8 assay showed that both parental and sunitinib-resistant RCC cell lines exhibited low sensitivity to SB-747651A (Figure 7O, S7L), whereas LDN-212854 demonstrated potent cytotoxicity with IC50 values below 1 µM, and notably, sunitinib-resistant cells were more sensitive to LDN-212854 compared with parental cells (Figure 7P, S7M). Treatment with LDN-212854 (0.5 μM) in combination with sunitinib (10 μM) produced a synergistic effect in TKI-resistant RCC cell lines (Figure S7N). mt-Keima assays further demonstrated that LDN-212854 suppressed sunitinib-induced mitophagy, leading to the accumulation of damaged mitochondria and subsequent cell death (Figure S7O).

## Discussion

**Figure 8.**
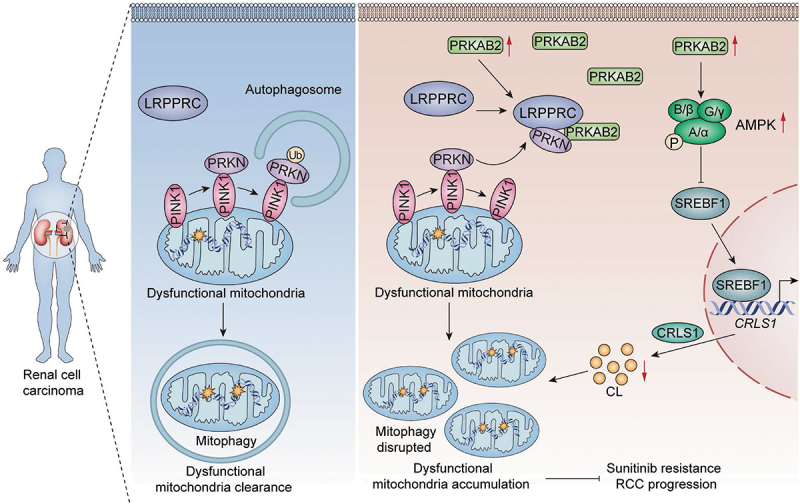
Proposed model of PRKAB2-mediated regulation of mitophagy and RCC progression. In RCC cells, PRKAB2 impairs mitophagy through two distinct mechanisms, leading to the accumulation of damaged mitochondria, leading to the accumulation of damaged mitochondria and consequently promoting RCC progression and resistance to sunitinib. On the one hand, PRKAB2 enhances the interaction between LRPPRC and PRKN, thereby reducing PRKN-PINK1 binding and suppressing the initiation of ubiquitin-dependent mitophagy. On the other hand, PRKAB2 activates PRKAA/AMPK phosphorylation, thereby suppressing the cleavage and nuclear translocation of SREBF1, which in turn diminishes its transcriptional activation of *CRLS1*, leading to reduced synthesis of cardiolipin, a key mitochondrial lipid essential for mitophagy, further compromising mitochondrial clearance. These dual mechanisms contribute to the accumulation of dysfunctional mitochondria, ultimately driving RCC progression and resistance to sunitinib.

In this study, we identified PRKAB2 as a critical tumor suppressor in RCC by performing an unbiased genome-wide CRISPR screen. Our findings indicate that PRKAB2 expression is significantly decreased in RCC tissues and cell lines. This downregulation is associated with increased tumor aggressiveness, advanced nuclear grading, and poor prognosis, suggesting a potential tumor-suppressive role of PRKAB2 in RCC. Mechanistically, PRKAB2 suppresses RCC progression by inhibiting mitophagy through dual regulation: enhancing LRPPRC-PRKN competitive binding to reduce PRKN-PINK1 interaction, and suppressing cardiolipin synthesis via AMPK-SREBF1-mediated downregulation of *CRLS1* (Figure 8). Moreover, we demonstrated that PRKAB2 could reverse TKIs resistance of RCC cells via inhibiting mitophagy flux, suggesting potential clinical implications for overcoming therapy resistance.

There has been limited research on PRKAB2 in cancer, and its role in tumor development and progression remains debated. In ovarian cancer, PRKAB2 has been observed to be significantly upregulated and potentially associated with mucinous and serous subtypes; however, its precise mechanism remains unclear [31]. Conversely, in colorectal cancer and pediatric adrenocortical tumors, PRKAB2 exhibits tumor-suppressive phenotype [32,33]. Our work provides the first direct evidence that PRKAB2 functions as a tumor suppressor in RCC. We demonstrated a consistent downregulation of PRKAB2 expression in RCC tumor samples compared to adjacent normal tissues. Moreover, lower PRKAB2 expression was significantly correlated with higher tumor grade, increased invasion, and shorter OS, underscoring its potential as a prognostic biomarker in RCC.

While these findings establish PRKAB2 as a tumor suppressor in RCC, the underlying molecular mechanisms remain largely unexplored. To address this, we investigated its functional role in key cellular processes and identified a novel PRKAB2-LRPPRC-PRKN axis regulating mitophagy. Previous studies on PRKAB2 have primarily focused on its role as a regulatory subunit of AMPK, implicating it in energy sensing and post-translational modifications such as SUMOylation and phosphorylation [8]. Additionally, *PRKAB1 PRKAB2* double knockout has been shown to reduce mitochondrial DNA content and oxidative enzyme activities [34], highlighting its importance in mitochondrial homeostasis. However, studies on AMPK-independent regulatory mechanisms remain scarce.

Through immunoprecipitation and GO analysis of mass spectrometry results, we identified a significant association between PRKAB2 and mitochondrial function, with LRPPRC emerging as the most prominent mitochondrial-associated protein interacting with PRKAB2. Given previous reports that LRPPRC inhibits mitophagy by directly binding to PRKN, we hypothesized that PRKAB2 May influence mitophagy in RCC through its interaction with LRPPRC [23]. To investigate this hypothesis, we examined the competitive binding dynamics between LRPPRC and PRKN, revealing that PRKAB2 overexpression significantly enhanced the LRPPRC-PRKN interaction. This, in turn, reduced PRKN’s binding with PINK1, thus suppressing the initiation of ubiquitin-dependent pathway of mitophagy. These findings suggest that PRKAB2 May function as a mitophagy modulator by shifting the equilibrium of PRKN’s binding partners, favoring its interaction with LRPPRC over PINK1.

While our data highlight a PRKAB2-specific mechanism involving LRPPRC-PRKN binding and AMPK-SREBF1-CRLS1 signaling, it remains possible that other AMPK subunits also regulate mitophagy through distinct molecular routes. For example, PRKAA subunits have been reported to phosphorylate MFF and ULK1 to regulate mitochondrial fission and autophagy initiation [35,36], and PRKAA1 and PRKAA2 have been implicated in mitophagy regulation under different stress conditions [37,38]. These studies suggest that although our results support a unique role of PRKAB2 in RCC, other AMPK isoforms may influence mitophagy in different biological contexts.

Notably, we also found that *LRPPRC* knockout only partially rescued the PRKAB2-induced phenotype, which prompted us to explore additional downstream pathways. Through this analysis, we uncovered a parallel lipid metabolic mechanism in which PRKAB2 suppresses cardiolipin synthesis via the AMPK-SREBF1-CRLS1 axis, thereby providing a complementary route to inhibit mitophagy.

Based on these findings, we propose a model in which PRKAB2 reinforces LRPPRC’s inhibitory role in mitophagy by promoting its sequestration of PRKN. This mechanism prevents PRKN recruitment to depolarized mitochondria, thereby suppressing mitophagy in RCC. Given the growing recognition of mitophagy as a key determinant of cancer cell survival and metabolic adaptation [39], our findings provide new insights into PRKAB2’s role in tumor progression beyond its established AMPK-related functions. These insights may also inspire therapeutic strategies, such as mimicking PRKAB2-induced conformational shift of LRPPRC or designing small-molecule inhibitors targeting PRKN’s PINK1-binding site to suppress mitophagy in RCC. Future studies should explore whether PRKAB2’s impact on mitophagy extends to other cancer types and how its regulation might be leveraged for therapeutic interventions.

Since *LRPPRC* knockout only partially reverses the tumor-suppressive phenotype driven by PRKAB2, we sought to investigate additional downstream pathways under its regulation. Indeed, the interplay between mitophagy and dysregulated lipid metabolism is likely to contribute to RCC tumorigenesis. Altered lipid metabolism is recognized as a hallmark of ccRCC metabolic adaptation. Initially driven by this insight, our study identified PRKAB2 as a candidate regulator in RCC based on *in vivo* CRISPR screening combining with lipid metabolism abnormalities. Subsequent targeted lipidomics analysis identified PRKAB2 as a crucial regulator of lipid homeostasis, significantly affecting triglyceride, cholesterol ester, and free fatty acid metabolism in RCC. Specifically, PRKAB2 overexpression substantially reduced cellular accumulation of these lipids, highlighting its pivotal role in RCC lipid metabolic modulation.

Unexpectedly, our findings revealed that PRKAB2 also inhibits mitophagy in RCC cells through its regulation on lipid biosynthesis. Cardiolipin, a critical phospholipid predominantly in the IMM, emerged as a central lipid mediator in mitochondrial quality control. Upon mitochondrial stress, cardiolipin translocates from the IMM to the OMM, serving as an “eat-me” signal to facilitate mitophagy. Specifically, externalized cardiolipin directly interacts with MAP1LC3 to initiate autophagosome formation [40] and induces mitochondrial fragmentation by activating the fission protein DNM1L, collectively facilitating mitochondrial degradation [22]. Currently, the regulation of mitophagy by cardiolipin has been reported to play a significant role in the occurrence and progression of malignant tumors such as hepatocellular carcinoma [41] and glioma [42].

Cardiolipin biosynthesis critically depends on two key enzymes, PGS1 and CRLS1. Our findings revealed that PRKAB2 negatively regulates mitophagy by suppressing cardiolipin biosynthesis via downregulation of these enzymes as well as PG, the precursor of cardiolipin [28] (Figure 6(M)). Consistent with this model, CRLS1 overexpression rescued PRKAB2-induced inhibition of mitophagy. Collectively, our findings establish a previously unrecognized regulatory axis linking PRKAB2-mediated lipid metabolic modulation with mitophagy in RCC, providing comprehensive mechanistic insights into the role of PRKAB2 beyond traditional AMPK signaling pathways. Targeting cardiolipin biosynthesis pathways may thus represent an innovative therapeutic strategy to modulate mitochondrial quality control and autophagic flux in RCC.

This study did not extensively explore the upstream regulation of PRKAB2. However, the research by Feng et al. [43] may provide some insights into this direction. They suggested that the expression of PRKAB2 is primarily transcriptionally activated through the TP53 pathway, thereby exerting downstream effects [43].

A critical clinical challenge in ccRCC therapy is the development of resistance to targeted therapies, particularly TKIs. Here, we demonstrated that enhanced mitophagy contributes to TKI resistance in RCC cells, as evidenced by elevated PRKN and MAP1LC3B-II expression in sunitinib-resistant cell lines. This observation is consistent with findings reported by Lu et al [7]. Notably, overexpression of PRKAB2 significantly reversed TKI resistance by suppressing excessive mitophagy flux, thereby restoring drug sensitivity. Conversely, increased PRKN expression, which promotes mitophagy, further exacerbated resistance to sunitinib and axitinib, underscoring mitophagy as a pivotal mechanism in drug resistance. Subsequent analysis utilizing the Depmap database further supported a negative correlation between TKI sensitivity and mitophagy activity. We also found that the sensitizing effect of PRKAB2 was more evident in resistant cells than in parental cells, likely because resistant cells exhibit elevated basal mitophagy, whereas parental cells maintain relatively low flux that is not strongly induced at non-lethal TKI concentrations. This context dependency suggests that PRKAB2 is particularly relevant in drug-resistant settings where mitophagy is pathologically upregulated.

This analysis also enabled us to identify LDN-212854 as a potential therapeutic candidate for cells with elevated mitophagy activity, which typically exhibit reduced responsiveness to TKIs. LDN-212854 targets ALK2 to inhibit BMP1 signaling and has been reported to suppress hepatocellular carcinoma [44]. However, most studies regarding LDN-212854 have primarily concentrated on its regulatory effects in ossification and osteoclastogenesis [45]. To date, no reports have directly linked LDN-212854 to mitophagy regulation or tumor resistance to TKIs. In this study, we explored the potential use of LDN-212854 as an adjunct in overcoming TKI resistance, while recognizing that the precise underlying mechanisms remain to be elucidated. Collectively, our findings provide a promising therapeutic strategy to overcome TKI resistance in RCC.

Here, our study presents several key innovations. First, we performed the first *in vivo* genome-wide CRISPR screening targeting RCC and identified PRKAB2 as a crucial regulator of RCC progression and TKI resistance, bridging metabolic reprogramming with mitophagy. Second, we uncovered a novel molecular mechanism by which PRKAB2 promotes the interaction between LRPPRC and PRKN, thereby reducing the binding of PRKN to PINK1, ultimately suppressing mitophagy and inhibiting RCC progression. Third, metabolomic analysis allowed us to elucidate that PRKAB2 regulates cardiolipin synthesis from a metabolic perspective, consequently impacting mitophagy and RCC progression. Lastly, we provided the first evidence of PRKAB2’s inhibitory effect on TKI resistance in RCC, identifying LDN-212854 as a promising therapeutic candidate for RCC cells exhibiting enhanced mitophagy flux and reduced TKI sensitivity, with *in vitro* validation demonstrating increased sensitivity of TKI-resistant cell lines to this compound.

Despite these advances, several limitations exist. Firstly, validation of PRKAB2 as a prognostic biomarker in larger clinical cohorts and prospective studies is necessary. Secondly, although our findings strongly support a PRKAB2-dependent mechanism, we did not directly assess whether other AMPK subunits may also contribute to mitophagy regulation in RCC. Future work will be required to determine whether the mitophagy-suppressive effect is unique to PRKAB2 or more broadly shared among AMPK isoforms. Furthermore, the specific regulation mechanisms of cardiolipin biosynthesis enzymes and their activity modulated by PRKAB2 need further exploration. Finally, translating these molecular findings into clinical applications requires additional preclinical validation, including *in vivo* efficacy studies using patient-derived xenograft models and clinical trials targeting mitophagy.

In conclusion, we conducted the first genome-wide *in vivo* CRISPR screen in RCC, identifying PRKAB2 as a key regulator linking lipid metabolism, mitophagy, and drug resistance. We demonstrated that PRKAB2 suppresses RCC progression by inhibiting PINK1-PRKN-mediated mitophagy through competitive binding of LRPPRC to PRKN. Additionally, PRKAB2 reduces cardiolipin content via lipid metabolism regulation, further restricting mitophagy. Functionally, PRKAB2 enhances TKI sensitivity in drug-resistant RCC cells by suppressing excessive mitophagy flux. Through targeted screening, we identified LDN-212854 as a potential treatment for high-mitophagy RCC cells. These findings highlight PRKAB2 as a promising therapeutic target for overcoming TKI resistance in RCC.

## Materials and methods

### Human RCC tissue samples

Matched RCC and adjacent non-tumorous renal tissue samples were collected from surgical resections at our institution. Samples were snap-frozen in liquid nitrogen and stored at −80°C immediately after excision. Prior to surgery, written informed consent was obtained from all participants, and the study was approved by the Ethics Committee of Huazhong University of Science and Technology ([2023]-0847).

### In vivo genome-wide screening and high-throughput sequencing analysis

The human genome-wide CRISPR/Cas9 knockout pooled library GeCKOv2 was obtained from GeneChem (Shanghai, China). 786-O cells were seeded at dish with polybrene. Lentiviruses were titrated to ensure an optimal virus concentration leading to approximately 30% cell survival. Cells were subjected to spinfection at 800 ×g for 2 h at 32°C, incubated overnight, trypsinized, pooled, and transferred into T225 flasks at a density of 3 × 10^6^ cells per flask. Puromycin (MedChemExpress, HY-B1743) selection (5 µg/mL) was performed for 7 days, after which 30 million cells were stored at −80°C for genomic DNA (gDNA) extraction and sequencing. The remaining cells were subcutaneously injected into NCG mice for *in vivo* screening.

### Genomic DNA extraction

Approximately 30 million cells or 200 mg of ground tumor tissues were lysed in 6 mL NK Lysis Buffer (50 mM Tris(hydroxymethyl)aminomethane, 50 mM ethylenediaminetetraacetic acid, 1% sodium dodecyl sulfate, pH 8) containing 30 µL proteinase K (20 mg/mL; Beyotime, ST535). Cell lysates were incubated at 55°C for 1 h (cells) or overnight (tissues). RNase A (final concentration 0.05 mg/mL; Beyotime, ST576) was added, followed by incubation at 37°C for 30 min. Samples were chilled on ice for 10 min, and then 2 mL of cold 7.5 M ammonium acetate (Beyotime, Y257425) was added, vortexed vigorously, and centrifuged at 4000 ×g for 10 min. Supernatants were mixed with isopropanol (Sinopharm, 80,109,218), precipitated DNA was washed with 70% ethanol (Sinopharm, 10,009,161), air-dried, and resuspended in 500 µL 1× TE Buffer (Beyotime, R0225) at 65°C for 1 h. DNA concentrations were quantified using an Ultramicro spectrophotometer (Kaiao, Beijing, China).

### sgRNA sequencing and data analysis

RNA-seq and subsequent analyses were performed in collaboration with Novagene (Beijing, China). DNA quality and potential contamination were evaluated via agarose gel electrophoresis and quantified with the Qubit system. Samples containing over 0.5 µg DNA were used for library preparation. Following end-repair and A-tailing, adapters were ligated, and PCR-free libraries were constructed. Libraries were first quantified using Qubit 2.0 (Thermofisher, MA, USA), validated for fragment size on an Agilent 2100 system (Agilent, CA, USA), and then precisely quantified by qPCR (3 nM). Qualified libraries were sequenced using the Illumina platform. Raw reads were processed for quality, adapters were trimmed, and low-quality reads were removed. Clean reads were mapped to the sgRNA reference library, and gene essentiality scores were calculated via MAGeCK’s Robust Rank Aggregation algorithm [46,47]. GO enrichment analysis was subsequently performed.

### Cell culture

RCC cell lines (ACHN, A-498, Caki-1, OS-RC-2, 786-O), HEK-293, and 293T cells (ACHN, American Type Culture Collection [ATCC], CRL-1611; A-498, ATCC, HTB-44; Caki-1, ATCC, HTB-46; OS-RC-2, National Collection of Authenticated Cell Cultures, TCHu 40; 786-O, ATCC, CRL-1932; HEK-293, ATCC, CRL-1573; 293T, ATCC, CRL-3216) were maintained in DMEM (Servicebio, G4515) supplemented with 10% FBS (Vazyme, F101-01) and 1% penicillin – streptomycin (Servicebio, G4003) under standard conditions (37°C, 5% CO_2_).

### Sunitinib-resistant cell line establishment

As previously described [48], sunitinib resistance was induced by gradually increasing drug concentrations. Resulting cell lines exhibited IC50 values exceeding twice those of their parental counterparts.

### Plasmid construction, transient transfection, and lentivirus infection assay

Plasmids for *PRKN* (Genomeditech, GM-MC-143872) and *CRLS1* overexpression (Genomeditech, GM-MC-146724) and LRPPRC truncations (GeneCreate, GS1-24080279) were transiently introduced into RCC cells at ~60% confluence using Lipofectamine 3000 (Invitrogen, L3000015). Lentiviral transduction for PRKAB overexpression and knockout of *PRKAB2* and *LRPPRC* was performed at ~40% confluence. Selection was carried out using puromycin (5 µg/mL; MedChemExpress, HY-B1743) or blasticidin (20 µg/mL; Solarbio, B9300). Knockout cells were plated into 96-well plates for single-cell cloning. Gene editing was verified by western blot analysis.

### Mice xenograft and metastasis models

All animal procedures were conducted with approval from the Animal Ethics Committee of Huazhong University of Science and Technology [IACUC Number 4659]. For xenograft models, 1 × 10^6^ ACHN cells were subcutaneously injected into BALB/c nude mice; for metastasis models, injections were performed via tail vein. Tumor size was recorded every 3 days using digital calipers. Tumor volume was estimated with the formula: V = (Length × Width^2^)/2. Metastatic spread was assessed using an in vivo imaging system (BRUKER, MA, USA), followed by tissue harvest and histological analysis. In drug resistance experiments, 3 × 10^6^ ACHN-R cells were subcutaneously implanted into 4-week-old nude mice. Starting from day 20 post-inoculation, mice received daily oral doses of sunitinib (60 mg/kg, MedChemExpress, HY-10255).

### Cell proliferation and drug sensitivity assays

Cell viability and sensitivity to sunitinib were assessed using the Cell Counting Kit-8 (CCK-8; MedChemExpress, HY-K0301). RCC cells were seeded into 96-well plates at a density of 1 × 10^3^ cells/well for proliferation assays and 5 × 10^3^ cells/well for drug response assays. For proliferation analysis, cell viability was recorded every 24 h over a 96-h period. For drug sensitivity testing, various concentrations of sunitinib were added after a 24-h incubation, followed by an additional 48-h culture. Subsequently, 10 μL of CCK-8 reagent was added per well and incubated for 2 h. Absorbance was measured at 450 nm using a microplate reader, and viability was calculated to determine proliferation rates and drug sensitivity.

### Three-dimensional cell culture and growth analysis

To assess cell growth under 3D conditions, RCC organoids were cultured in Matrigel (Corning, 356,234). Cells were dissociated into single-cell suspensions at 500–1,000 cells/mL and mixed with Matrigel at a 1:1 volume ratio. A 30 µL aliquot of the mixture was plated in the center of each well in a 48-well plate and incubated at 37°C to allow gelation. Culture medium was then added, and plates were maintained at 37°C with 5% CO_2_. Media were refreshed every 3 days. Organoid development was monitored by microscopy, and images were captured at regular intervals.

### Cell migration and invasion assays

Transwell chambers were used to evaluate the migratory and invasive capacities of RCC cells. Prior to the assay, cells were serum-starved in DMEM for 6–8 h. For migration assays, 3 × 10^4^ cells were seeded into uncoated upper chambers; for invasion assays, 6 × 10^4^ cells were plated into chambers pre-coated with Matrigel. The lower chambers contained DMEM supplemented with 10% FBS as a chemoattractant. After 6 h of incubation at 37°C, cells that had migrated or invaded to the lower membrane surface were fixed with paraformaldehyde (Servicebio, G1101), stained using 0.1% crystal violet (Servicebio, G1014), rinsed with PBS (Servicebio, G4202), and imaged. Migrated or invaded cells in five randomly selected fields per chamber were counted under a microscope.

### Lipid staining and quantification assays

RCC cells were fixed in paraformaldehyde, rinsed with PBS, and stained with Oil Red O (Servicebio, G1015) for 30 min at room temperature. Excess stain was removed by gentle PBS washing, and lipid droplets were visualized under light microscopy. Intracellular levels of cholesterol and triglycerides were quantified using commercially available kits (Jiancheng, A111-1–1 and A110-1–1), following the manufacturer’s instructions.

### RNA isolation and quantitative RT-PCR

Total RNA was extracted from RCC tissues and cultured cells using the Ultrapure RNA Extraction Kit (CWBIO, CW0599S). First-strand cDNA was synthesized using the HiScript II Reverse Transcriptase (Vazyme, R201-01). Quantitative real-time PCR was performed using SYBR-Green Master Mix (Vazyme, Q311-02) on a Bio-Rad detection system (Hercules, CA, USA). Primers for gene amplification were synthesized by TSINGKE Biotechnology (Beijing, China), with sequences listed in Table S4.

### Western blotting

Proteins were isolated from RCC tissues and cells using RIPA buffer (Beyotime, P0013) containing phenylmethanesulfonyl fluoride (Beyotime, ST506) and protease inhibitors (Servicebio, G2006). For analysis of phosphorylated proteins, phosphatase inhibitors (Servicebio, G2007) were also included. Protein samples (30 μg per lane) were resolved via sodium dodecyl sulfate-polyacrylamide gel electrophoresis and transferred to polyvinylidene difluoride membranes (Roche, 03010040001). After blocking, membranes were incubated with primary antibodies, followed by secondary antibody incubation for 1.5 h. Antibodies against ACTB (20536–1-AP), LRPPRC (67679–1-Ig), p-ACACA (29119–1-AP), PRKN (14060–1-AP), PGS1 (17149–1-AP), PINK1 (23274–1-AP), PTPMT1 (11493–1-AP) were purchased from Proteintech. Antibodies against BNIP3 (A19593), CRLS1 (A12388), DNM1L/DRP1 (A21968), FUNDC1 (A22001), HA-Tag (AE008, AE036), HMGCR (A1633), MAP1LC3B (A11282), LRPPRC (A3365), MFN1 (A21293), MFN2 (A19678), MYC-Tag (AE010, AE070), BNIP3L (A24803), p-PRKAA (AP0432), PRKAA1 (A16960), PRKAA2 (A7339), PRKAB1 (A4344), PRKAB2 (A6952), UCP1 (A5857) were purchased from Abclonal. Antibodies against PRKAG/AMPKγ (PA5813) and FLAG (M20118, R20008) were purchased from Abmart. Antibody against PRKAB2 (sc-376752) was purchased from Santa Cruz Biotechnology.

### IHC and Hematoxylin-Eosin (H&E) staining

Technical support was from Biossci (Wuhan, China). Paraffin-embedded patient and xenograft tumor tissues were deparaffinized, rehydrated, and subjected to antigen retrieval using either EDTA (0.01 M, pH 9.0) or citrate buffer (0.01 M, pH 6.0). Endogenous peroxidase activity was blocked with 3% hydrogen peroxide (Sinopharm, 10,011,218) for 30 min. Sections were then incubated overnight at 4°C with primary antibodies, followed by incubation with secondary antibodies at 37°C for 45 min. DAB (MXB Biotechnology, DAB4033) was used for visualization, and nuclei were counterstained with hematoxylin (Biossci, BP0211). For H&E staining, tissues were stained with hematoxylin, differentiated, briefly counterstained with eosin (Biossci, BP0211), and dehydrated. Images were acquired using a DSZ2000 microscope (UOP Photoelectric Technology, Chongqing, China). Antibody against PRKAB2 (14429–1-AP) was purchased from Proteintech. Antibody against LRPPRC (A3365) was purchased from Abclonal.

### Immunofluorescence staining

For cellular immunofluorescence, RCC cells (1 × 10^5^) were seeded onto sterile round coverslips (Biosharp, BS-18-RC), fixed with 4% paraformaldehyde for 10 min, permeabilized using 0.5% Triton X-100 (Biosharp, BS084) for 10 min, and blocked with 5% BSA (Servicebio, GC305010). Coverslips were incubated overnight at 4°C with primary antibodies (MAP1LC3B [Abclonal, A11282], LRPPRC [Abclonal, A3365], PRKN [Proteintech, 14,060–1-AP], PRKAB2 [Abclonal, A6952], TOMM20 [Proteintech, 66,777–1-Ig]), followed by 2-h incubation at room temperature with fluorophore-conjugated secondary antibodies (ABflo 594 goat anti-mouse IgG [ABclonal, AS054] or ABflo 488 donkey anti-rabbit IgG [ABclonal, AS035]; 1:250). Nuclei were stained using DAPI (Beyotime, C1002), and images were acquired with a Nikon AX confocal microscope (Tokyo, Japan). Multiplex immunofluorescence staining with tyramide signal amplification was supported by Biossci (Wuhan, China), and image capture was performed at Jarvisbio (Wuhan, China).

Tissue-based immunofluorescence utilizing tyramide signal amplification was conducted on paraffin sections. The protocol included deparaffinization, rehydration, antigen retrieval (citrate buffer, pH 6.0 or EDTA buffer, pH 9.0), and sequential application of primary and secondary antibodies. Tyramide signal amplification reagents were applied for 10 min following antibody binding. Between staining rounds, antigen-antibody complexes were eluted at 42°C. Nuclei were counterstained with DAPI, and fluorescence images were obtained using the DSZ2000 microscope (UOP Photoelectric Technology, Chongqing, China). Primary antibody information is summarized above.

### Transmission electron microscopy imaging

TEM imaging was conducted with assistance from Servicebio (Wuhan, China). Cells were harvested by centrifugation, resuspended in fixative, and stored at 4°C. After embedding in agarose (Servicebio, GC205013), samples were post-fixed in 1% osmium tetroxide (Ted Pella, 18,456) in phosphate buffer (0.1 M, pH 7.4, Servicebio, G4202) at room temperature for 2 h, followed by three rinses in the same buffer. Specimens were dehydrated through graded ethanol and acetone series, then infiltrated with increasing concentrations of EMBed 812 resin (SPI Supplies, 90,529–77-4) and polymerized at 60°C for at least 48 h. Ultrathin sections (60–80 nm) were mounted on 150-mesh Formvar-coated copper grids (Servicebio, WFHM-150), stained sequentially with 2% uranyl acetate (SPI Supplies, 02624-AB) and 2.6% lead citrate (Sigma-Aldrich, 203,580), and visualized using a Hitachi HT7800 transmission electron microscope (Tokyo, Japan).

### Co-immunoprecipitation

Co-IP was carried out following previously described protocols [12]. RCC cells were lysed using RIPA buffer (Beyotime, P0013), and lysates were incubated overnight at 4°C with constant rotation in the presence of primary antibodies or isotype-matched IgG controls. Protein A/G magnetic beads (MedChemExpress, HY-K0202) were then added for 4 h at room temperature to capture immune complexes. After washing with PBST (PBS with 0.05% Tween-20 [Beyotime, ST825]) to eliminate nonspecifically bound proteins, complexes were eluted and analyzed via western blotting.

### Sructure prediction and binding interface analysis

Three-dimensional structural predictions of protein complexes were generated using AlphaFold’s multimer model [49]. Amino acid sequences of each subunit were input simultaneously, and the resulting structural models were ranked based on AlphaFold’s internal confidence scores. The highest-confidence model was selected for downstream analysis. Protein-protein interaction interfaces were analyzed using PyMOL v3.0.3. Solvent-accessible surface areas (SASA) were computed for each subunit alone and for the assembled complex. The interface area (ΔSASA) was calculated by subtracting the SASA of the complex from the sum of individual subunit SASAs, representing the buried surface upon complex formation. Hydrogen bonds at the interface were identified based on donor – acceptor distances ≤3.5 Å and visualized as dashed lines. Residues contributing significantly to interface stability, either through extensive buried surface area or multiple hydrogen bonds, were considered key contact sites.

### Broad-spectrum targeted lipidomics analysis

Lipidomic profiling was performed with support from Wuhan Metware Biotechnology Co.,Ltd. Cells stored at −80°C were thawed on ice, and lipids were extracted using 1 mL of methyl t-butyl ether (ThermoFisher, E127-4): methanol (Merck, 1.06007.4008) (3:1, v:v) containing internal standards. Samples were vortexed for 15 min, mixed with ultrapure water, vortexed again for 1 min, and centrifuged at 14,800 ×g or 10 min. The upper organic phase was collected, dried in a vacuum concentrator, and reconstituted in acetonitrile (Merck, 1.00030.4008):isopropanol (Merck, 1.01040.4008) (1:1, v:v) prior to Liquid Chromatography-Tandem Mass Spectrometry (LC-MS/MS) analysis.

Lipid detection was carried out on an LC-ESI-MS/MS system (UPLC, ExionLC AD; MS, QTRAP 6500+, Sciex) with separation on a Thermo Accucore™ C30 column (Thermo, 27,826). A binary solvent system was used: solvent A (acetonitrile:water, 60:40) and solvent B (acetonitrile:isopropanol, 10:90), both containing 0.1% formic acid (Sigma-Aldrich, F112034-500 ml) and 10 mM ammonium formate (Fisher, A115-50). The flow rate was 0.35 mL/min at 45°C. Data were acquired in both positive and negative ESI modes with optimized multiple reaction monitoring transitions. Differential lipid species were identified via Orthogonal Projections to Latent Structures-Discriminant Analysis (VIP > 1, *p* < 0.05) using MetaboAnalystR with log transformation and permutation tests (200 iterations). Kyoto Encyclopedia of Genes and Genomes and Metabolite Set Enrichment Analysis databases were used for metabolite identification and pathway enrichment, with statistical significance assessed using hypergeometric testing.

### Liquid chromatography-tandem mass spectrometry (LC-MS/MS) analysis

Proteomic profiling was conducted by Novogene Co., Ltd (Beijing, China) using an EASY-nLC™1200 UHPLC system coupled with either a Q Exactive™ HF-X or Orbitrap Exploris 480 mass spectrometer (Thermo Fisher, Germany). Peptides (1 µg) were loaded onto a self-packed C18 nano-trap column (4.5 cm × 75 µm, 3 µm; Bio-Rad, 1,633,007). Elution was performed using a gradient of solvent A (0.1% formic acid [ThermoFisher, A117-50] in water) and solvent B (0.1% formic acid in 80% acetonitrile [ThermoFisher, A955-4]). The 40 most abundant peptide ions were fragmented by high-energy collisional dissociation and subjected to Tandem Mass Spectrometry (MS/MS). MS scans were acquired over m/z 350–1500 with a resolution of 60,000 and 20 ms injection time; MS/MS scans were set at 15,000 resolution. Raw data were analyzed using Proteome Discoverer with parameters: precursor mass tolerance of 10 ppm, product ion tolerance of 0.02 Da, fixed carbamidomethylation, and variable modifications including methionine oxidation and N-terminal methionine loss. Protein identification required at least one unique peptide, with peptide-spectrum matches filtered at ≥99% confidence and FDR ≤1%.

### Cardiolipin detection

Cardiolipin content was quantified using a commercial ELISA kit (Meimian, MM-2281H1) following the manufacturer’s protocol.

### Bioinformatics analysis

Gene expression profiles (FPKM) and associated clinical data were retrieved from TCGA (https://portal.gdc.cancer.gov/, accessed August 2022) and GEO (GSE46699). Lipid metabolism-related gene sets were downloaded from the Molecular Signatures Database (Table S1). Protein-protein interaction networks were constructed using STRING and visualized with Cytoscape. Key hub genes were identified using the CytoHubba plugin based on multiple topological parameters, including Betweenness, Bottleneck, Closeness, Clustering coefficient, degree, dnnc, EcCentricity, epc, mcc, nnc, Radiality, stress [50].

Drug sensitivity data and transcriptomic profiles of cancer cell lines were obtained from the DepMap portal (https://depmap.org/). Pearson correlation analysis was used to assess gene-drug relationships. Mitophagy scores were calculated via Gene Set Variation Analysis in R (v4.4.1), based on the GOBP_MITOPHAGY gene set from the Molecular Signatures Database.

### Statistical analyses

Kaplan-Meier survival analyses and log-rank tests were used to compare OS between groups stratified by gene expression levels. OS was defined as the time from diagnosis to death or last follow-up. Statistical analyses were performed using R (v4.4.1) and GraphPad Prism (v9.4.1), with Student’s t-test, one-way ANOVA, or nonlinear regression applied as appropriate. All experiments were conducted independently at least three times.

## Supplementary Material

Supplementary table 4.xlsx

Supplementary table 2.xlsx

Supplementary_Figure_PRKAB2_R3.docx

Supplementary table 1.xlsx

Supplementary table 3.xlsx

## Data Availability

The datasets supporting the conclusions of this manuscript are included within the manuscript and supporting information. Other data and materials about this study are available from the corresponding author on reasonable request.
