# Supplementary material for: PRKAB2 as a tumor suppressor in renal cell carcinoma: inhibiting mitophagy via the LRPPRC-PRKN/parkin interaction and cardiolipin biosynthesis
Source: Autophagy. 2026 Feb 18;22(5):982–1002. doi: 10.1080/15548627.2026.2623985 (PMC13232994; doi:10.1080/15548627.2026.2623985)
Supplement: Supplementary_Figure_PRKAB2_R3.docx [file KAUP_A_2623985_SM5988.docx]

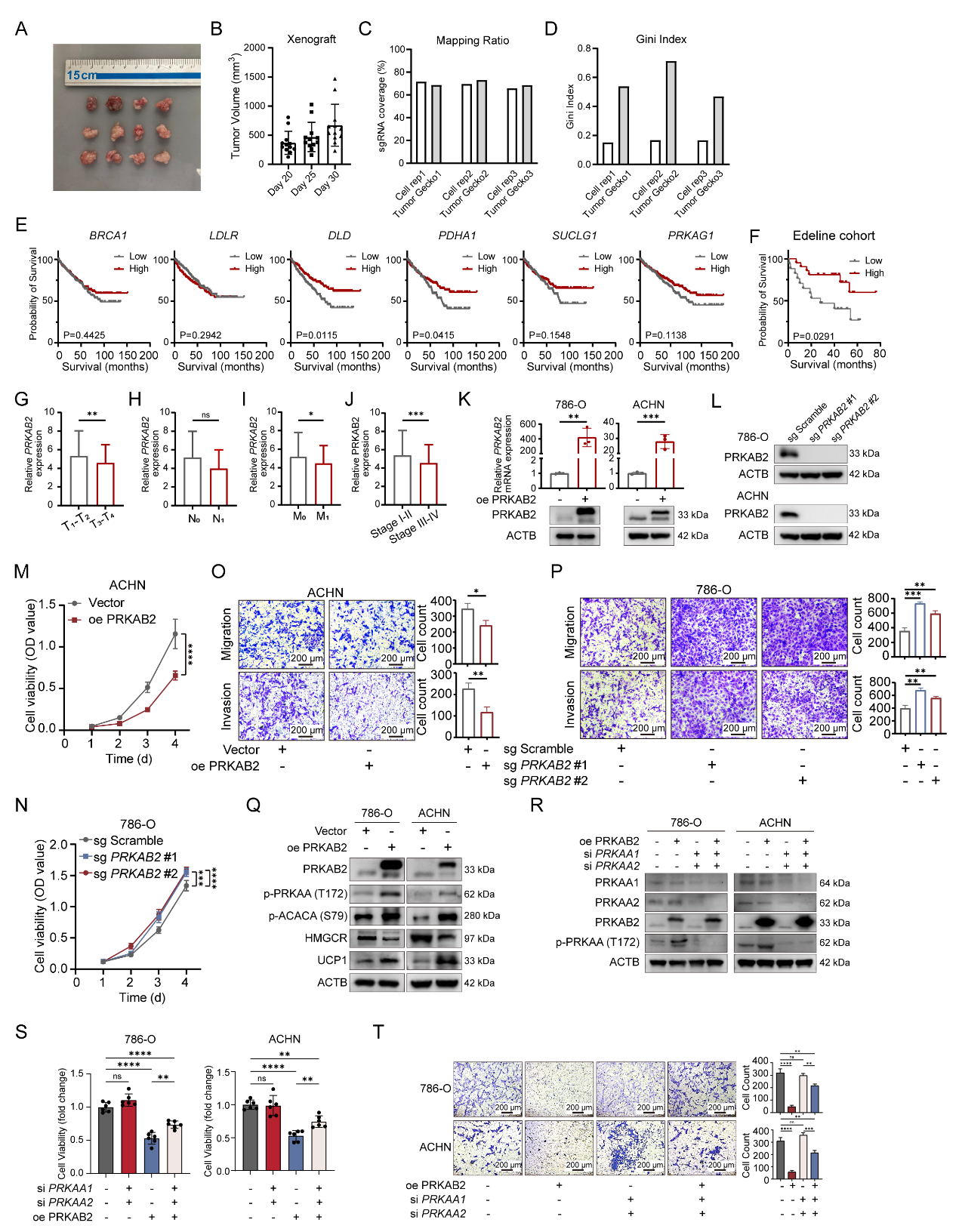


**Figure S1.** Characterization of genome-wide CRISPR screening and validation of PRKAB2 function in RCC. (**A**) Representative images of xenograft tumors from 786-O cells infected with the genome-wide CRISPR library. (**B**) Tumor volume changes in xenografts derived from 786-O cells infected with the genome-wide CRISPR library. (**C**) Mapping ratio analysis of sgRNAs from CRISPR high-throughput sequencing. (**D**) Gini index analysis of sgRNA distribution in CRISPR high-throughput sequencing. (**E**) Kaplan-Meier survival analysis of RCC patients with high versus low expression of BRCA1, LDLR, DLD, PDHA1, SUCLG1, and PRKAG1 in the TCGA-KIRC dataset. (**F**) Kaplan-Meier survival analysis of RCC patients with high versus low PRKAB2 expression in the Edeline cohort. (**G**) PRKAB2 expression analysis in RCC tumor tissues from patients with T_1_-T_2_ versus T_3_-T_4_ disease stages in the TCGA-KIRC dataset. (**H**) PRKAB2 expression analysis in RCC tumor tissues from patients with N_0_ versus N_1_ lymph node metastasis status in the TCGA-KIRC dataset. (**I**) PRKAB2 expression analysis in RCC tumor tissues from patients with M_0_ versus M_1_ metastatic status in the TCGA-KIRC dataset. (**J**) PRKAB2 expression analysis in RCC tumor tissues from patients with stage I-II versus stage III-IV disease in the TCGA-KIRC dataset. (**K**) PCR and Western blot analysis confirming PRKAB2 overexpression in 786-O and ACHN cells. (**L**) Western blot analysis confirming *PRKAB2* knockout in 786-O and ACHN cells using the CRISPR-Cas9 system. (**M and N**) CCK-8 assay assessing the effect of PRKAB2 overexpression on ACHN cell proliferation (M) and *PRKAB2* knockout on 786-O cell proliferation (N). (**O and P**) Transwell assay evaluating the impact of PRKAB2 overexpression on ACHN cell invasion and migration (O) and *PRKAB2* knockout on 786-O cell invasion and migration (P). (**Q**) Western blot analysis of PRKAA phosphorylation and downstream lipid-related markers (p-ACC1, HMGCR, UCP1) in vector and PRKAB2 overexpressing RCC cells. (**R**) Western blot analysis of PRKAA1 and PRKAA2 knockdown efficiency and PRKAA phosphorylation in RCC cells. (**S**) CCK-8 assay assessing the proliferative capacity of RCC cells following PRKAB2 overexpression and/or *PRKAA1* and *PRKAA2* silencing. (**T**) Transwell assay assessing the migration capacity of RCC cells following PRKAB2 overexpression and/or *PRKAA1* and *PRKAA2* silencing. * P < 0.05; ** P < 0.01; *** P < 0.001; ns, not statistically significant.


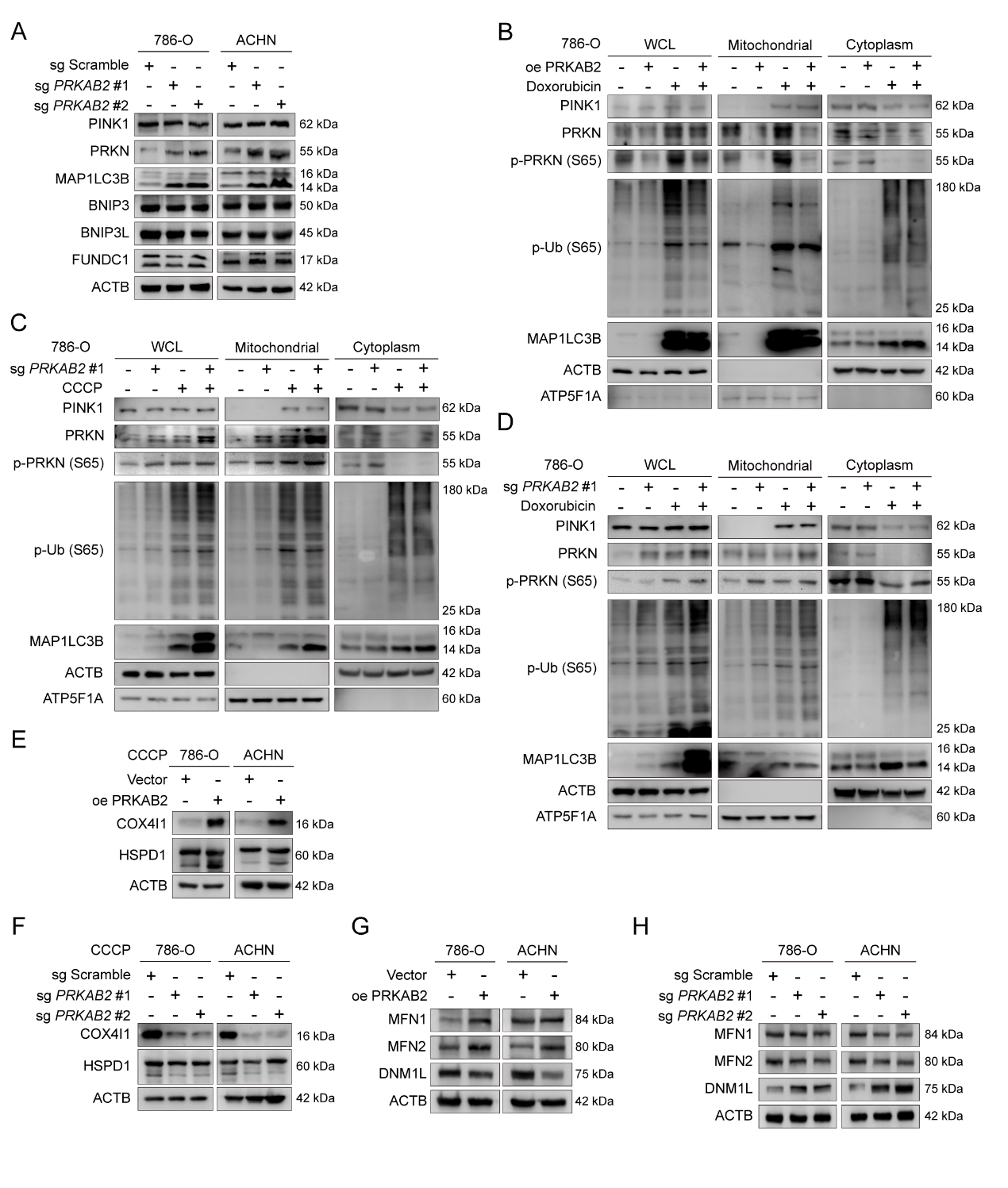
 **Figure S2.** PRKAB2 regulates mitophagy and mitochondrial dynamics in RCC cells. (**A**) Western blot analysis of mitophagy-related markers in RCC cells following *PRKAB2* knockout. (**B**) Western blot analysis of mitophagy-related markers in mitochondrial, cytosolic, and WCL fractions of RCC cells, as indicated. (**C and D**) Western blot analysis of mitophagy-related markers in mitochondrial, cytosolic, and WCL fractions of *PRKAB2* knockout RCC cells under pressure from CCCP (C) or doxorubicin (D). (**E and F**) Western blot analysis of mitochondrial content-related proteins in *PRKAB2* overexpressing (E) or knockout (F) cells. (**G and H**) Western blot analysis of fusion- and fission-related proteins in *PRKAB2* overexpressing (G) or knockout (H) cells.


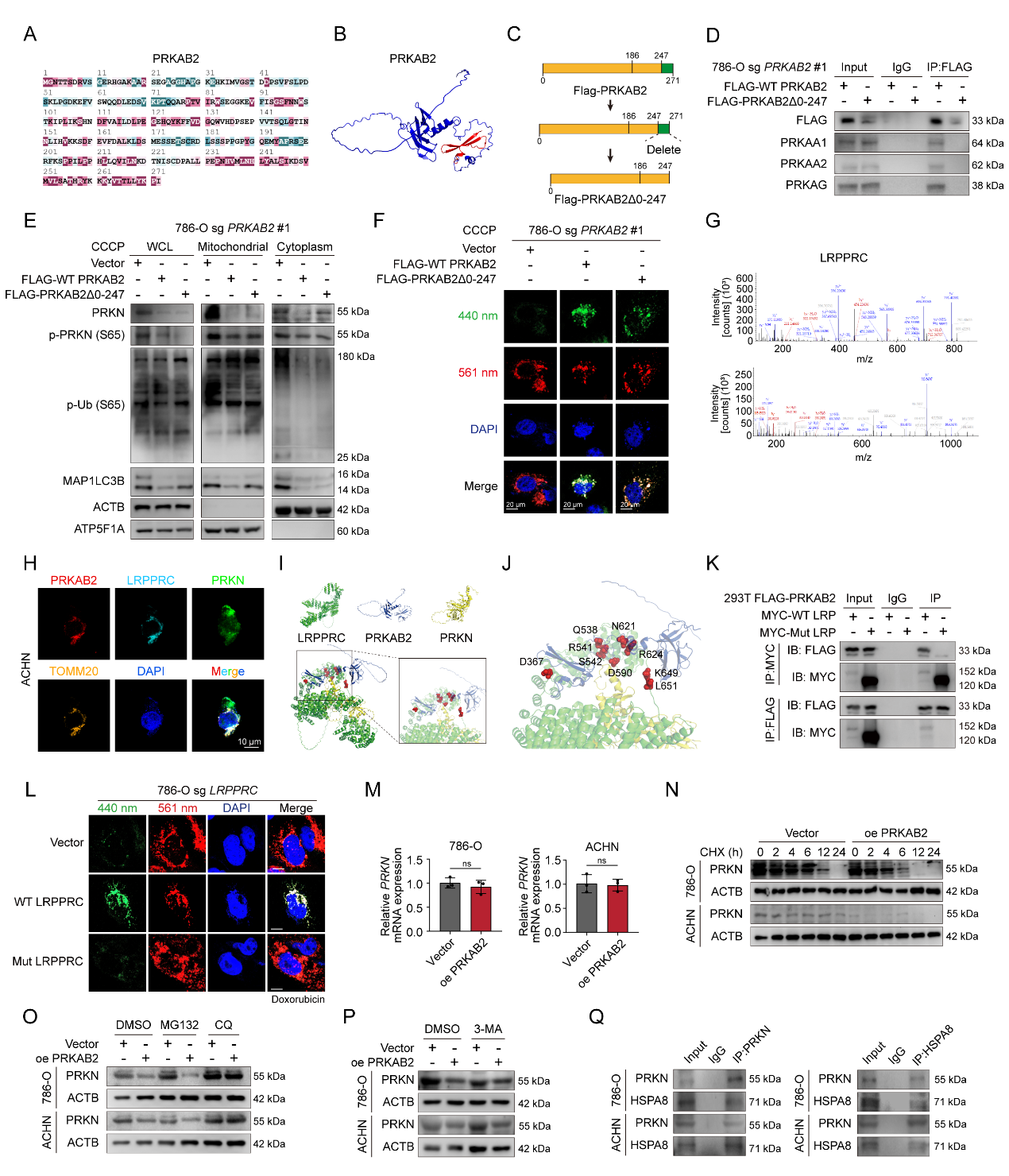
 **Figure S3.** Functional analysis of PRKAB2 and LRPPRC mutants and the role of chaperone-mediated autophagy in mitophagy regulation. (**A**) Sequence conservation analysis of PRKAB2. (**B**) Predicted three-dimensional structure of the conserved C-terminal region of PRKAB2 generated using AlphaFold and PyMOL. (**C**) Schematic of PRKAB2 mutant (labeled as FLAG-PRKAB2Δ0-247) sequences deficient in AMPK complex assembly. (**D**) Co-IP and western blot analysis validating the ability of WT- and PRKAB2∆0-247 to assemble into the AMPK complex. (**E**) Western blot analysis of mitophagy-related markers in mitochondrial, cytosolic, and WCL fractions of RCC cells, as indicated. (**F**) mt-Keima assay assessing mitophagy in 786-O cells following treatment with 10 μM CCCP for 2 h. Fluorescence channels: 440 nm (neutral pH) and 561 nm (acidic pH). scale bar: 20 μm. (**G**) Mass spectrum of LRPPRC identified in LC-MS/MS Analysis. (**H**) Immunofluorescence staining in ACHN cells to assess the subcellular localization of PRKAB2, LRPPRC, PRKN, and TOMM20. scale bar: 10 μm. (**I**) AlphaFold and PyMOL predictions of hydrogen bond interactions at the LRPPRC-PRKAB2 binding interface. (**J**) Predicted key amino acid residues at the LRPPRC-PRKAB2 binding interface, based on AlphaFold and PyMOL structural modeling. (**K**) Co-IP and western blot analysis validating the ability of WT and mutant LRPPRC to bind PRKAB2. MYC-WT LRP: refer to MYC-WT LRPPRC; MYC-Mut LRP: refer to MYC-Mut LRPPRC. (**L**) mt-Keima assay assessing mitophagy in 786-O cells following treatment with Doxorubicin. Fluorescence channels: 440 nm (neutral pH) and 561 nm (acidic pH). scale bar: 20 μm. (**M**) qPCR analysis of *PRKN* mRNA in vector and PRKAB2 overexpressing RCC cells. (**N**) Cycloheximide chase assay assessing PRKN protein stability. CHX: cycloheximide. (**O**) Western blot analysis of PRKN levels in PRKAB2 overexpressing cells treated with chloroquine or MG132. CQ: chloroquine. (**P**) Western blot analysis of PRKN levels following 3-MA treatment. 3-MA: 3-methyladenine. (**Q**) Co-IP analysis of PRKN interaction with HSPA8. * P < 0.05; ** P < 0.01; *** P < 0.001; ns, not statistically significant.


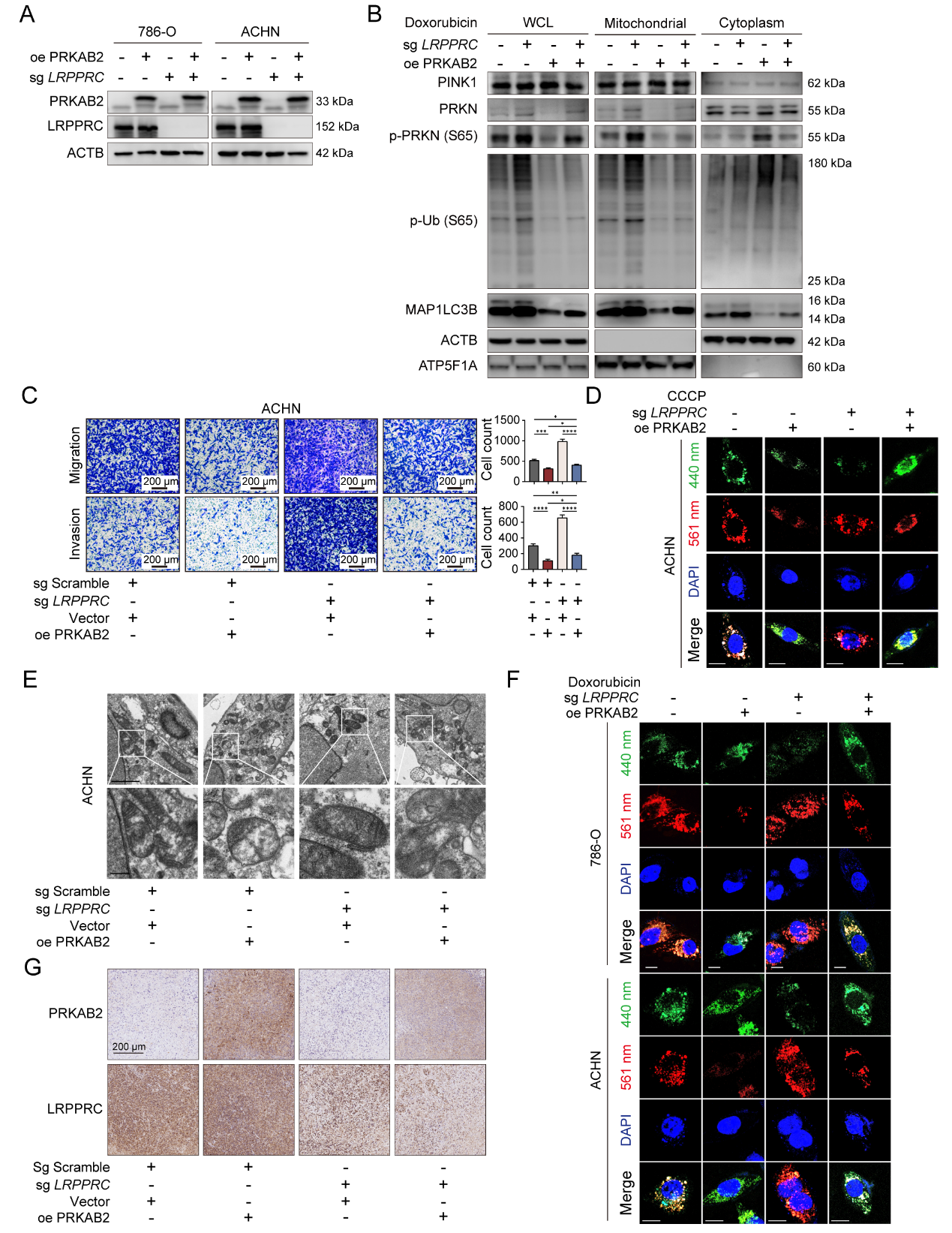
 **Figure S4.** PRKAB2 overexpression and *LRPPRC* depletion regulate RCC cell migration, invasion, and mitophagy. (**A**) Western blot analysis of PRKAB2 and LRPPRC expression in RCC cells as indicated. (**B**) Mitochondrial fractionation and western blot analysis of ubiquitin-dependent mitophagy markers in RCC cells following treatment with Doxorubicin. (**C**) Transwell invasion and migration assays in ACHN cells, as indicated. (**D**) mt-Keima assay assessing mitophagy in ACHN cells following treatment with 10 μM CCCP for 2 h. Fluorescence channels: 440 nm (neutral pH) and 561 nm (acidic pH). scale bar: 20 μm (**E**) Transmission electron microscopy analysis of mitochondrial damage in ACHN cells following treatment with 10 μM CCCP for 2 h, as indicated. scale bars: 1 μm (low magnification), 500 nm (high magnification). (**F**) mt-Keima assay assessing mitophagy in 786-O and ACHN cells following treatment with CCCP or Doxorubicin. Fluorescence channels: 440 nm (neutral pH) and 561 nm (acidic pH). scale bar: 20 μm. (**G**) Immunohistochemistry staining of xenograft tumors as indicated. scale bar: 200 μm. * P < 0.05; ** P < 0.01; **** P < 0.0001; ns, not statistically significant.


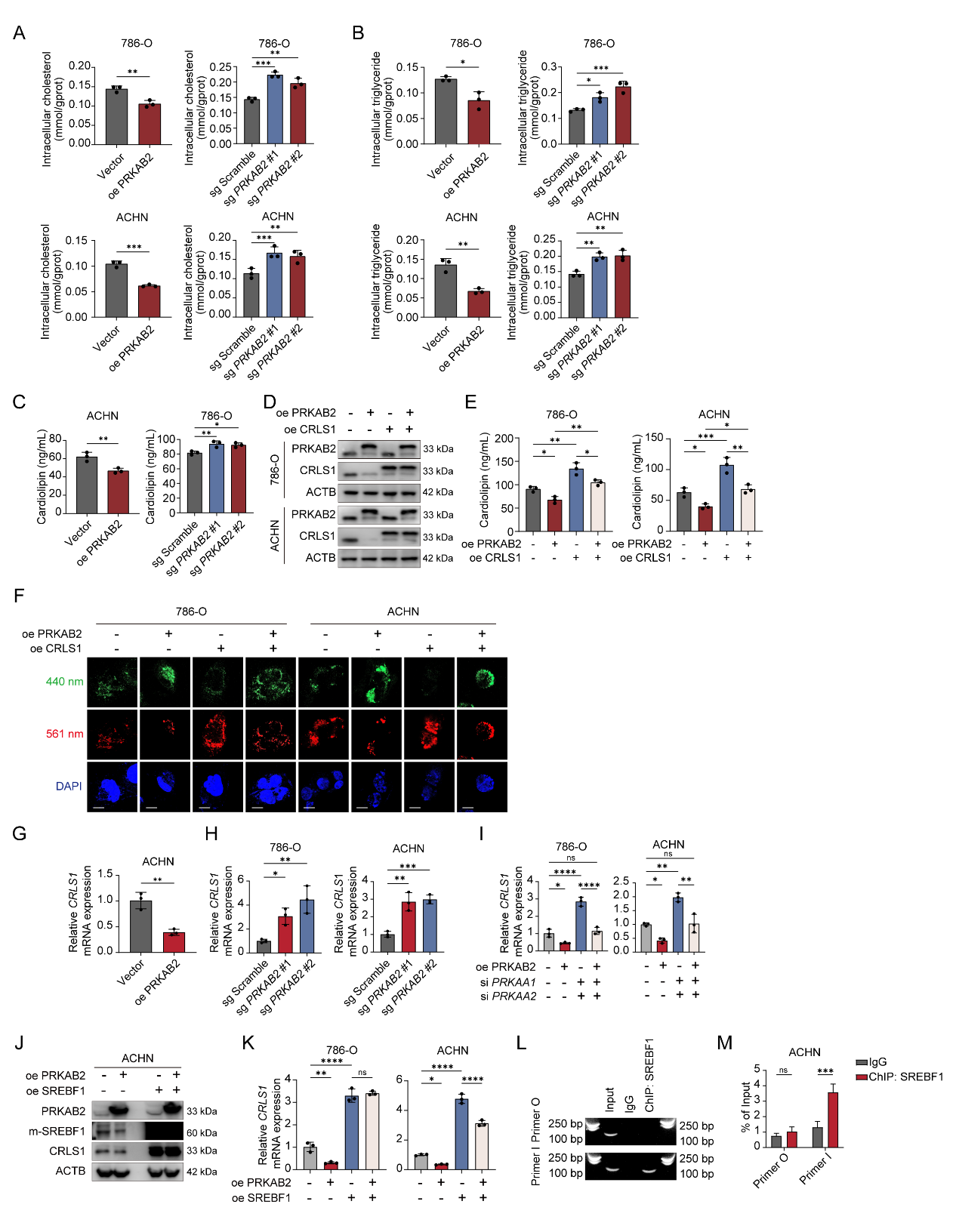
 **Figure S5.** PRKAB2-mediated regulation of lipid metabolism in RCC cells. (**A and B**) Quantification of intracellular cholesterol (A) and triglyceride levels (B) in RCC cells with *PRKAB2* overexpression or knockout. (**C**) Quantification of intracellular cardiolipin levels in RCC cells, as indicated. (**D**) Western blot analysis of PRKAB2 and CRLS1 expression levels in RCC cells, as indicated. (**E**) Quantification of intracellular cardiolipin levels in RCC cells, as indicated. (**F**) mt-Keima assay assessing mitophagy in RCC cells following treatment with 10 μM CCCP for 2 h. Fluorescence channels: 440 nm (neutral pH) and 561 nm (acidic pH). scale bar: 20 μm. (**G**) qPCR analysis of *CRLS1* mRNA in vector and PRKAB2-overexpressing ACHN cells. (**H**) qPCR analysis of *CRLS1* mRNA in vector and *PRKAB2* knockout RCC cells. (**I**) qPCR analysis of *CRLS1* mRNA in RCC cells as indicated. (**J**) Western blot analysis of CRLS1 protein expression levels, as indicated. (**K**) qPCR analysis of *CRLS1* mRNA in RCC cells as indicated. (**L**) DNA gel electrophoresis showing the amplification products of SREBF1 ChIP with different primers, as indicated. (**M**) qPCR analysis of amplified products from SREBF1 ChIP using different primer sets. * P < 0.05; ** P < 0.01; *** P < 0.001; ns, not statistically significant.


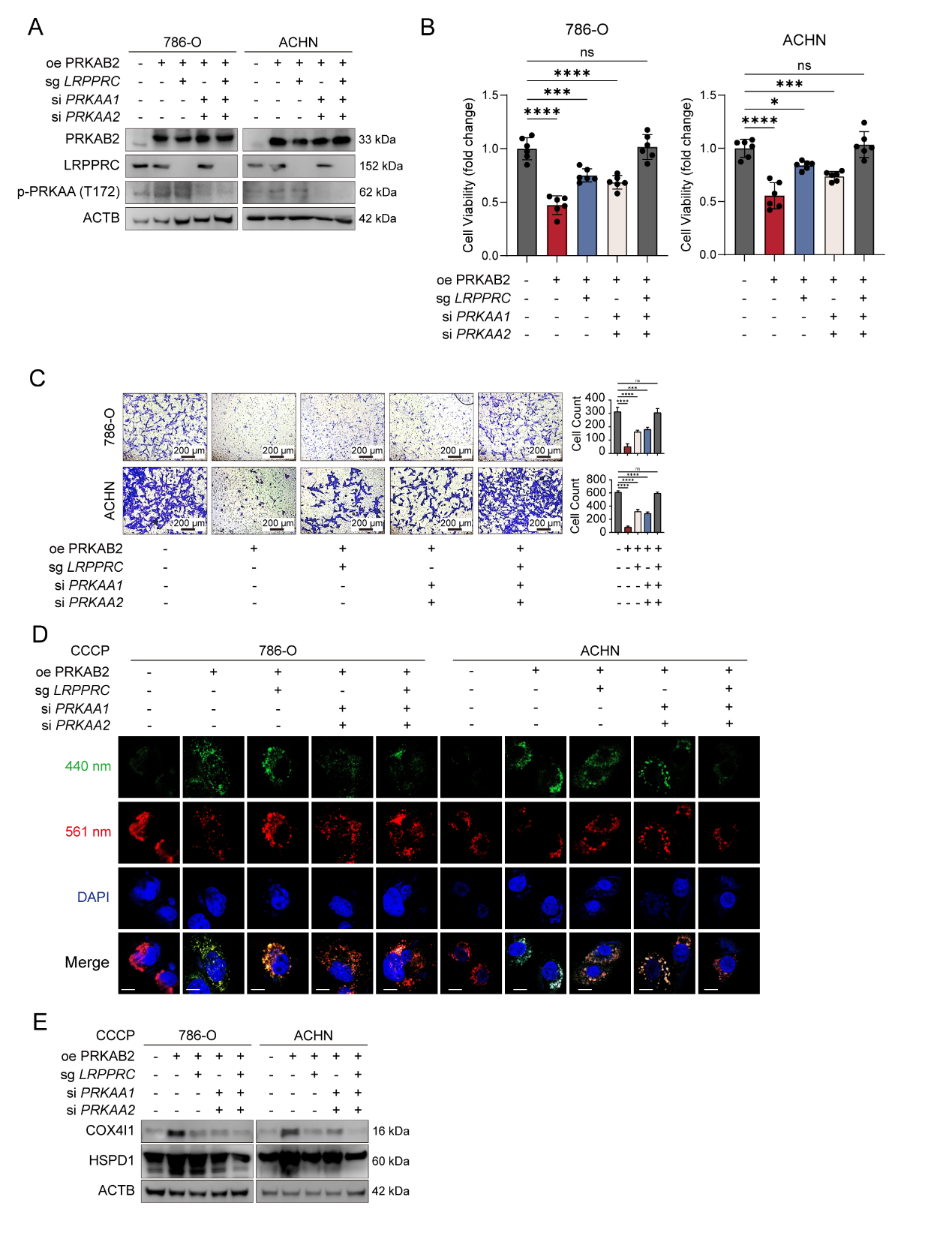
 **Figure S6.** Simultaneous *LRPPRC* knockout and *PRKAA1* and *PRKAA2* silencing completely reversed the inhibitory effects of PRKAB2 on both mitophagy and RCC progression. (**A**) Western blot analysis of PRKAB2 and LRPPRC expression in RCC cells as indicated. (**B**) CCK-8 assay assessing the proliferative capacity of RCC cells, as indicated. (**C**) Transwell assay assessing the migration capacity of RCC cells, as indicated. (**D**) mt-Keima assay assessing mitophagy in RCC cells following treatment with 10 μM CCCP for 2 h. Fluorescence channels: 440 nm (neutral pH) and 561 nm (acidic pH). scale bar: 20 μm (**E**) Western blot analysis of mitochondrial content-related proteins in RCC cells, as indicated. * P < 0.05; ** P < 0.01; *** P < 0.001; ns, not statistically significant.


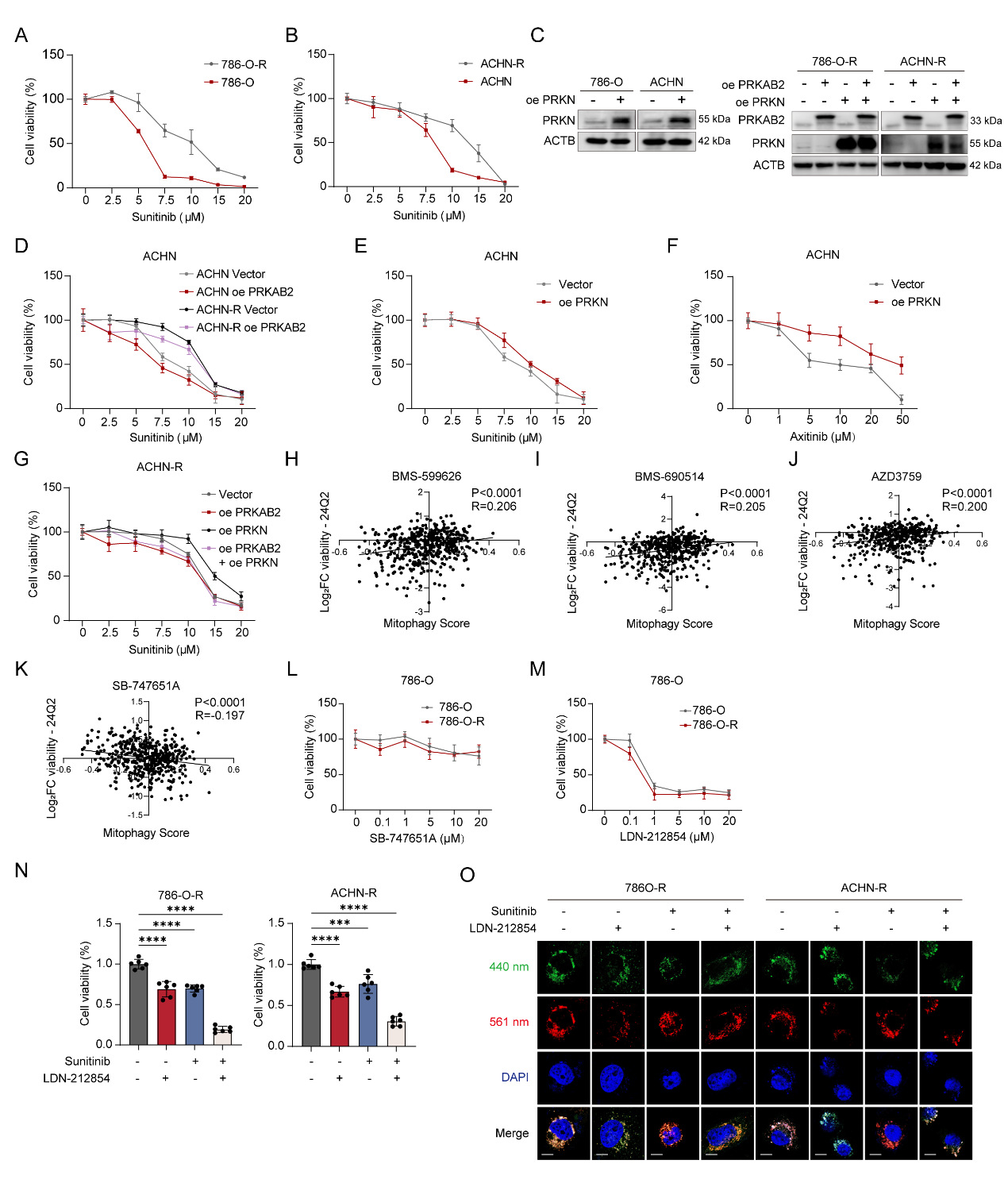


**Figure S7.** PRKAB2 modulates mitophagy and therapeutic responses to TKIs in RCC. (**A and B**) CCK-8 assay measuring sunitinib IC50 in parental and sunitinib-resistant 786-O (A) and ACHN (B) cell lines. (**C**) Western blot validation of PRKAB2 and PRKN overexpression in parental and sunitinib-resistant RCC cell lines. (**D**) CCK-8 assay measuring sunitinib IC50 in parental and sunitinib-resistant ACHN cells overexpressing PRKAB2. (**E and F**) CCK-8 assay measuring IC50 values of sunitinib (E) and axitinib (F) in ACHN cells overexpressing PRKN. (**G**) CCK-8 assay measuring sunitinib IC50 in sunitinib-resistant ACHN cells overexpressing PRKAB2 or PRKN. (**H-J**) Scatter plots from DepMap database showing correlations between mitophagy scores and drug sensitivity for BMS-599626 (H), BMS-690514 (I), and AZD3759 (J). (**K**) Scatter plots from DepMap database showing correlations between mitophagy scores and drug sensitivity for SB-747651A. (**L and M**) CCK-8 assay measuring IC50 values of SB-747651A (L) and LDN-212854 (M) in parental and sunitinib-resistant 786-O cells. (**N**) CCK-8 assay assessing cell viability under different drug concentrations, as indicated. (**O**) mt-Keima assay assessing mitophagy in TKI resistant RCC cells following different treatment, as indicated. Fluorescence channels: 440 nm (neutral pH) and 561 nm (acidic pH). Scale bar: 20 μm * P < 0.05; ** P < 0.01; *** P < 0.001; ns, not statistically significant.
